# An overview of reviews of breastfeeding barriers and facilitators: Analyzing global research trends and hotspots

**DOI:** 10.1016/j.gloepi.2025.100192

**Published:** 2025-03-06

**Authors:** Agustín Ramiro Miranda, Paula Eugenia Barral, Ana Veronica Scotta, Mariela Valentina Cortez, Elio Andrés Soria

**Affiliations:** a MoISA, University of Montpellier, CIRAD, CIHEAM-IAMM, INRAE, Institut Agro, IRD, 911 Avenue d'Agropolis, Cedex 5, 34394 Montpellier, France; b Universidad Nacional de Córdoba, Facultad de Ciencias Médicas, Bv. de la Reforma, Ciudad Universitaria, 5014 Córdoba, Argentina; c Consejo Nacional de Investigaciones Científicas y Técnicas, INICSA, Bv. de la Reforma, Ciudad Universitaria, 5014 Córdoba, Argentina

**Keywords:** Breastfeeding, Lactation, Barriers, Facilitators, Bibliometrics, Research trends, Science mapping

## Abstract

Breastfeeding is the most cost-effective intervention for reducing infant morbidity and mortality, offering benefits for infants and mothers. Despite extensive promotion, global adherence remains below 50 %, resulting in significant clinical, economic, and environmental impacts. Thus, this overview of reviews aims to synthesize barriers and facilitators of breastfeeding, analyze research trends, and identify gaps to guide future research. A comprehensive literature search was conducted, including systematic reviews that examine these factors. The search covered seven electronic data repositories. The methodological quality was assessed using the Risk of Bias in Systematic Reviews tool. Bibliometric analysis focused on identifying top journals, authors, and countries, assessing their impact, and exploring trends over time. Findings were classified and analyzed thematically through line-by-line coding, theme description, and analytical formulation. A total of 123 reviews were included, mostly of high quality and published in top journals. Key trends comprised a growing focus on psychosocial and cultural factors, increased representation from low- and middle-income countries, and improved methodological rigor. However, geographical representation remains biased towards high-income countries, and some breastfeeding outcomes need further exploration. Thematic analysis revealed four categories: Therapeutic and care interventions; Support networks and education; Maternal-infant health issues; and Societal and environmental context. In conclusion, this overview of reviews identifies barriers and facilitators of breastfeeding and emphasizes the need for more inclusive research and tailored support. Addressing gaps in evidence for enhancing healthcare systems and policies can improve breastfeeding practices and outcomes worldwide.

## Introduction

Human milk is the most appropriate source of nutrients and bioactive compounds for newborns, supporting their psycho-emotional development [1]. This biological fluid plays a crucial role in the health of both infants and mothers, with positive outcomes throughout the lifespan. According to the World Health Organization (WHO), breastfeeding should be exclusive until six months and mixed until at least two years of age [2], constituting one of the most cost-effective public health strategies to improve child health and survival [3]. Children who are breastfed for longer periods show lower morbidity and mortality from infectious diseases, dental issues, long-term overweight, and diabetes, as well as better cognitive development [4]. Additionally, breastfeeding benefits maternal health, acting as a protective factor against oncological, metabolic, cardiovascular, and neurodegenerative diseases in adulthood [5].

Over time, considerable efforts have been made to promote breastfeeding through health policies to achieve international standards. Notable among these initiatives are the WHO International Code of Marketing of Breastmilk Substitutes (1981), the joint WHO and UNICEF statement “Protecting, promoting, and supporting breastfeeding” (1982), the Innocenti Declaration on the protection, promotion, and support of breastfeeding (1990), and the Baby-Friendly Hospital Initiative (1992) [6]. Despite the benefits of breastfeeding and these international initiatives, less than half of the world's children are currently breastfed according to recommendations [3]. In response to this reality, the WHO has set a goal to achieve a 50 % adherence rate by 2025 [7]. Various factors influence the initiation and continuation of breastfeeding, including socioeconomic aspects, the absence of human milk in the first hour of life, and limited access to education, among others [4,8].

The lack of adherence to breastfeeding recommendations has multiple clinical, economic, and environmental consequences. It is estimated that adherence could save more than 820,000 infant lives annually and represent savings of over $300 billion per year [3,4]. Additionally, 20,000 breast cancer deaths could be avoided, with the number being even higher if the preventing effects on other pathologies were considered (e.g., cardiometabolic diseases) [3,4,9]. Besides the lives saved, the economic impact is significant, as economic models estimate that 0.49 % of the global gross product is lost due to the lack of universal breastfeeding [10]. For every dollar invested in breastfeeding support, an economic return of $35 has been estimated, related to reduced infant morbidity and mortality and improved cognitive outcomes [11].

Human milk is a sustainable food with an almost zero ecological and carbon footprint, requiring only 500 additional calories and one liter of potable water per day [12]. In contrast, formula milk has a greater environmental impact, consuming numerous resources [13]. For example, breastfeeding a child for the first six months could save approximately 22.4 kg of powdered milk, 105,280 l of water, and 488 kg of carbon dioxide equivalents [12].

In this context, promoting breastfeeding plays a central role in contributing to the Sustainable Development Goals (SDGs) [14]. This United Nations initiative includes 17 goals to be achieved by 2030, aiming to link sustainability with social inclusion and address the needs of the most vulnerable populations [15]. Increasing adherence to breastfeeding would favor several SDGs, such as poverty end (SDG 1), zero hunger (SDG 2), good health and well-being (SDG 3), quality education (SDG 4), reducing inequalities (SDG 10), sustainable development (SDG 11), and climate action (SDG 13) [14]. To meet the 2030 agenda targets, a multifaceted approach is needed that strengthens socio-technical transitions towards sustainable development and innovation, while sensitizing various stakeholders [16]. In this sense, breastfeeding is a social responsibility to which everyone can contribute and requires urgent attention [17].

However, to improve adherence to the established recommendations, it is essential to understand their determinants [18]. According to the WHO, adherence is the degree to which a person's behavior corresponds with the agreed recommendations from healthcare professionals concerning health-related activities such as taking medication, following a diet, or modifying lifestyle habits [19]. Understanding the causes of not adhering to breastfeeding, its determinants, and its effects would help address underlying issues and improve the effectiveness of interventions, including promotion and prevention programs. It would also allow health managers to continuously evaluate recommendations and make decisions to modify them or adjust communication styles to increase adherence. Failing to align the evaluation of the costs and benefits of health interventions with the preferences of the target population can result in adherence levels significantly lower than expected [20].

Multiple factors influencing the peripartum period and breastfeeding adherence have been identified, including physical factors (maternal and neonatal pathologies), mental factors (anxiety, postpartum depression, and insomnia), social factors (education level, income, and gender roles), and health system factors (quality of healthcare practices), which must be addressed [21]. A comprehensive overview of the determinants of breastfeeding can serve as a solid foundation for developing future interventions. Thus, the objectives of this overview of reviews are to: 1) synthesize the barriers and facilitators reported in previous reviews that applied systematic methods; 2) examine research activity through a bibliometric analysis; and 3) identify knowledge gaps to guide future priorities. Consequently, this overview of reviews aims to answer the following research question: What are the main barriers and facilitators influencing breastfeeding adherence and outcomes?

## Methods

This overview of reviews was conducted in accordance with the PRIOR (Preferred Reporting Items for Overviews of Reviews) guidelines (Supplementary Materials 1) and the recommendations of the Joanna Briggs Institute (JBI) Umbrella Review Methodology Working Group [22,23]. A public search protocol was registered on the Open Science Framework (OSF) under DOI: 10.17605/OSF.IO/TMS26. No amendments were made to the protocol (Supplementary Materials 2).

### Search strategy

The search was performed in the data repositories PubMed, Scopus, Cochrane Library, BMC Systematic Reviews, LILACS, and Health Evidence, and the research platform EBSCOhost (including the databases Academic Search Premier; Art, Education, Education Index Retrospective; Environment Complete; Academic Search; Humanities & Social Sciences Index Retrospective; Humanitiest, Library Literature & Information Science; Library Literature & Information Science Retrospective; Psychology & Behavioral Sciences Collection; Social Sciences; and SocINDEX, as licensed to the Ministry of Science, Technology, and Innovation of Argentina). Keywords, detailed in Table 1, were used in combination with boolean operators (OR/AND) to conduct comprehensive searches within titles, abstracts, subject headings (e.g., Medical Subject Headings [MeSH]), and other relevant search fields. Details on the search strategies performed on each data repository are provided in Table S1 (Supplementary Materials 3). Additionally, manual searches were performed on the reference lists of selected review articles for potentially eligible articles. The search spanned the entire history of each database and was updated until April 30, 2024.

**Table 1:** Search strategy for PubMed.

Search query	Keywords (searched within all fields)
1	((newborn) OR (neonate) OR (infant)) AND ((postpartum woman) OR (postnatal woman) OR (puerperal woman) OR (lactating woman))
2	((breastfeeding) OR (lactation) OR (breast feeding) OR (chestfeeding))
3	((determinants) OR (barriers) OR (facilitators) OR (factors) OR (inequities) OR (disparities) OR (beliefs) OR (myths))
4	((discontinuation) OR (cessation) OR (interruption) OR (termination) OR (duration) OR (latching) OR (self-efficacy) OR (failure) OR (initiation) OR (supply) OR (sucking) OR (suction) OR (suckling) OR (bonding) OR (attachment))
Filters	(meta-analysis OR systematic review)
Final search query	1 AND 2 AND 3 AND 4 AND Filters

### Inclusion and exclusion criteria

A modified PICOS strategy was used to formulate a focused research question consisting of P (population), I (phenomena of interest), C (context), O (outcome), and S (study) [24]. In this sense, studies were eligible for inclusion if they satisfied the following criteria:

1) Participants/population: The study population comprised lactating individuals, with no restrictions on socio-demographic factors, health status, or reproductive characteristics.
2) Phenomena of interest: The review aimed to identify barriers and facilitators affecting (any) breastfeeding among lactating individuals. Barriers and facilitators were defined as the factors that either hinder or promote a woman's ability to initiate and sustain breastfeeding, respectively [10,17]. These factors can be biological, psychological, social, or structural, which are studied across individual, community, and policy levels.
3) Context/setting: The review addressed any type of care setting, including community, primary healthcare, or acute care, as well as any living or geographical environment.
4) Outcomes: The review reported associations between barriers and facilitators with breastfeeding outcomes, such as duration, cessation, discontinuation, exclusive breastfeeding, and self-efficacy.
5) Types of studies: included studies were review articles published in peer-reviewed journals that employed defined methodologies for literature searching, specifically systematic reviews, meta-analyses, and systematic scoping reviews. These systematic evidence syntheses follow structured approaches that use reproducible methods to gather data from primary research, critically evaluate its quality, and synthesize findings descriptively or quantitatively. Key elements include a clear research question, detailed search strategies, inclusion and exclusion criteria, study selection methods, bias assessments, and robust data analysis procedures, ensuring reproducibility and reliability of the results.

No restrictions were applied regarding language or publication date. Articles were excluded if they failed to meet any of the specified criteria.

### Screening and selection of the literature

All citations identified from database searches and additional sources were imported into Rayyan® [25], a cloud-based platform designed for screening citation data in systematic reviews. After duplicates were removed, the “blind on” function was activated to ensure that collaborators could not view each other's decisions and labels during the screening process. Two authors (ARM and PEB) independently screened all citations using the review's inclusion and exclusion criteria. Any discrepancies identified during this initial screening were resolved through discussion with a third author (AVS). Subsequently, the full texts of the preliminary selected articles were reviewed to assess their eligibility, and any articles not meeting the specified criteria were excluded.

### Data extraction and analysis

Data was extracted from the final selection of articles using a prepiloted manual data extraction form using Google Forms (Supplementary Materials 2). Two authors (ARM and PEB) independently extracted data on the following domains: the country of affiliation of the corresponding author, the titles and objectives of the reviews, the type of systematic review method used, the databases searched, any language or time restrictions applied, the types of primary studies included in the reviews, the countries of origin of those studies, sample sizes, barriers and/or facilitators analyzed, breastfeeding outcomes reported, significant findings between barriers/facilitators and breastfeeding outcomes, methodological quality and risk-of-bias assessment tools, quality of included primary articles, and recommendations. All included reviews were assessed for potential data discrepancies. If discrepant data were observed, authors planned to retrieve the primary studies for analysis. However, no discrepant data were identified. The journal impact factor and quartiles of journals at the time of publication were obtained from www.scijournal.org and www.scimagojr.com, respectively. For each article, the total number of citations in Web of Science and the citation density (defined as the total number of citations in Web of Science/years since publication) were also computed [26].

Due to the heterogeneity in methods, population characteristics, breastfeeding outcomes, and barriers/facilitators across the reviews, a narrative synthesis was conducted. Additionally, trends were statistically assessed using SPSS (version 30) and Stata (version 18) through Bayesian one-way ANOVA (with a tolerance of 0.000001 and 20,000 samples from the Markov-Chain Monte Carlo - MCMC) and Bayesian ordered regression (with 10,000 MCMC samples and 2500 burn-in samples), using non-informative priors. Results were expressed as means (M) and 95 % credibility intervals (95 % CI) for the posterior distribution of each coefficient.

### Assessment of methodological quality

The methodological quality and risk of bias of the included reviews was assessed using the ROBIS (Risk of Bias in Systematic Reviews) tool [27]. This instrument includes three phases: phase 1 assesses the relevance of the review; phase 2 identifies concerns with the review process, subdivided into four domains (study eligibility criteria [D1], identification and selection of studies [D2], data collection and study appraisal [D3], and synthesis and findings [D4]); and phase 3 evaluates the overall risk of bias in the review [27]. Phase 1 is optional and was not performed in this study. Each question in the ROBIS tool offered five possible answers: “yes,” “probably yes,” “probably no,” “no,” and “no information,” with scores assigned as 3, 2, 1, or 0, respectively. A scoring system with defined cut-off values was devised to evaluate the methodological quality. For the first three ROBIS domains, each with five items, scores ranged from 0 to 15 and were categorized as low (0–4), moderate (5–9), or high (10–15). The ROBIS-D4 domain, which included six items, had a total score range of 0 to 18 and was categorized as low (0–5), moderate (6–11), or high (12–18). The ROBIS-Bias domain, consisting of three items, yielded scores from 0 to 9 and was categorized as low (0–3), moderate (4–6), or high (7–9).

Two authors (PEB and AVS) independently conducted the quality assessment, while a third author (ARM) double-reviewed 44 % of the studies. This process resulted in the following weighted Cohen's kappa coefficients: ROBIS-D1 = 0.97, ROBIS-D2 = 0.90, ROBIS-D3 = 0.82, ROBIS-D4 = 0.88, and ROBIS-Bias = 0.87. All coefficients exceeded 0.80, indicating almost perfect agreement according to standard approaches [28].

### Synthesis methods

The data analysis followed a three-step thematic synthesis approach [29], aimed at maintaining a clear and transparent connection between the conclusions and reviewed texts. This method involves: 1) line-by-line coding of findings; 2) organizing these codes into related areas to create descriptive themes; and 3) developing analytical themes. The result sections of 123 publications were coded line by line, with the codes and corresponding sentences recorded in a Microsoft 365 Excel worksheet to track content, meaning, and quotes. The thematic synthesis process was conducted collaboratively in virtual meetings, with all authors working together to organize the codes into descriptive themes. All authors have diverse expertise in breastfeeding research: one medical doctor specialized in epidemiology and maternal mental health, another in epidemiology and lactation in workplace settings, and a third one in pharmacology and breastfeeding medicine. Additionally, a nutritionist with a focus on maternal and child nutrition and a speech and language therapist with expertise in gender and social determinants of breastfeeding provided further specialized insights. The team also included two authors with personal breastfeeding experience.

All bibliometric text data extracted from databases were analyzed using VOSviewer version 1.6.19 [30]. To assess the strength of connections between terms appearing in titles and abstracts, Total Link Strength (TLS) was utilized, a metric automatically calculated by VOSviewer during the analysis. TLS reflects the strength of relationships, with higher values indicating stronger connections between items. For network mapping, a minimum of five occurrences for each term was established. Structured abstract subheadings (such as introduction, objective, methods, results, and conclusions), copyright statements, and unconnected items were excluded from the analysis and network visualization. The full counting method, which assigns equal weight to each link, was applied to texts in the title and abstract fields to generate a term co-occurrence network map.

## Results

### Search results

**Fig. 1.**
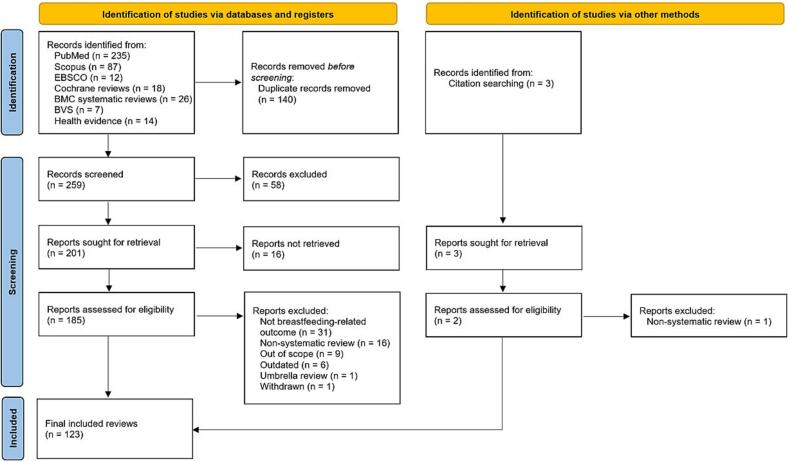
Flow chart of retrieved, screened and included articles.

The literature search process used to identify systematic reviews is illustrated in Fig. 1. From seven electronic databases, a total of 399 articles were identified, all of which were written in English. After removing duplicates, 259 articles were selected for the initial phase of title and abstract screening. Following this first phase, 201 reviews were selected for full-text evaluation, although access could not be obtained for 16 of them. During the second phase, which involved full-text reading, 62 reviews were excluded for the following reasons: not reporting results on breastfeeding (*n* = 31), not following a systematic methodology (*n* = 16), being outside the scope of this study's objectives (*n* = 9), being outdated (i.e., having an updated version, *n* = 6), being an overview of reviews (n = 1), or having been withdrawn (n = 1). Finally, after removing one non-systematic review, we added two more systematic reviews that were found through manual searches of reference lists. In total, 123 reviews were included in this study: 121 from the databases and two identified through manual reference searches. The characteristics of the retrieved reviews are detailed in the Supplementary Materials 3 (Table S2), and the list of articles excluded at the full-text stage is presented in Table S3.

### Journals and article citation

**Fig. 2.**
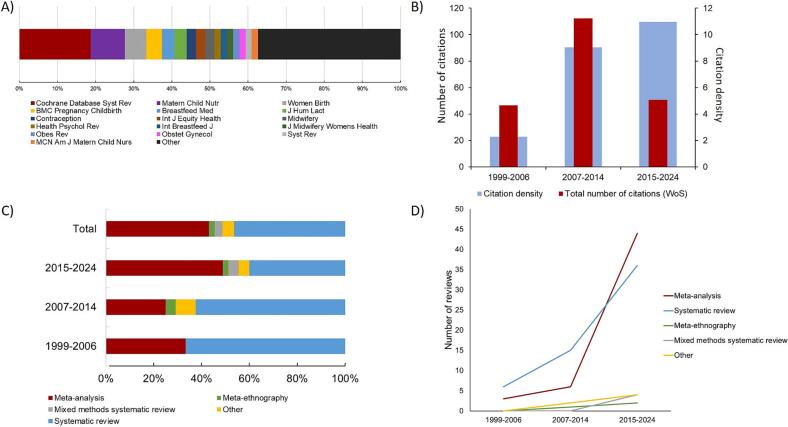
Publications by journal sources (A), impact of retrieved reviews (B), and design used (proportion in C, absolute number in D) across years.

The articles were published in 62 different journals, with 16 journals publishing at least two articles, representing 62.6 % of the articles (Fig. 2A). The top five most active journals were Cochrane Database of Systematic Reviews (18.7 %, 2051 citations), Maternal and Child Nutrition (8.9 %, 385 citations), Women and Birth (5.7 %, 438 citations), BMC Pregnancy and Childbirth (4.1 %, 568 citations), and Breastfeeding Medicine (3.3 %, 469 citations). In total, the top five journals published 40.7 % of reviews and received 3911 citations that accounted for 51.0 % of the total citations.

Regarding journal metrics, the majority of articles (approximately 80 %) were published in Q1 journals, regardless of the specialty, and this proportion remained constant over the years. On the other hand, the impact factor of the journals at the time of publication of the reviews increased over the periods, rising from 1.68 (95 %IC = 0.30–3.06) in the period 1999–2006 to 3.65 (95 %IC = 3.22–4.09) in the period 2015–2024 (Fig. S1).

The years of publication with the highest total number of citations ranged from 2007 to 2014. However, the period 2015–2024 exhibited the highest citation density (Fig. 2B). Positive trends were observed in both the total number of citations and citation density. The total number of citations increased from 8.09 (95 % CI = −146.57 to 162.75) in the 1999–2006 period to 52.61 (95 % CI = 3.70 to 16.87) in the 2015–2024 period. Similarly, citation density rose from 2.89 (95 % CI = −12.95 to 162.75) in 1999–2006 to 10.96 (95 % CI = 6.35 to 15.57) in 2015–2024.

### Study design used by reviews

In terms of literature review design, 46.3 % of the studies were systematic reviews, while 43.1 % were meta-analyses (Fig. 2C). Meta-ethnographic studies comprised 2.4 % of the publications, whilst 3.3 % were mixed-methods systematic reviews. The remaining 4.9 % involved other types of systematic literature reviews, such as systematic scoping reviews. Over time, the number of publications involving meta-analyses, meta-ethnographies, mixed-methods systematic reviews, and other systematic review methodologies increased noticeably (Fig. 2D).

### Most productive countries (based on corresponding authors)

The corresponding authors of these publications were from 25 different countries, with 19 countries contributing only one or two articles each. The country with the highest number of publications is the United States of America (USA) with 34 published reviews (27.6 %), followed by the United Kingdom (UK, *n* = 30, 24.4 %), Australia (*n* = 15, 12.2 %), Canada (*n* = 10, 8.1 %), China (*n* = 6, 4.9 %), and Switzerland (n = 3, 2.4 %) (Supplementary Materials 3, Fig. S2).

### Methodological quality of reviews

**Fig. 3.**
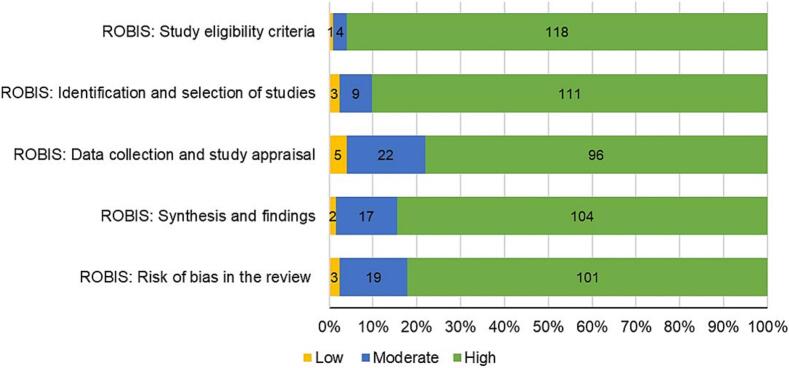
Methodological quality and risk of bias assessment of the retrieved reviews.

Fig. 3 displays the results of the methodological quality assessment of publications. Overall, most studies (>80 %) exhibited high quality when evaluated using the ROBIS tool. Moreover, there was an increase in the methodological quality of the publications over time across all domains (Table 2). Upon examining these dimensions, the lowest scores were related to issues with data collection, study appraisal, and risk of bias in the reviews. Furthermore, Bayesian ordered regression was used to assess the relationship between quality and time period, considering the ordinal nature of the variables, with mean Bayesian coefficients ranging from 0.85 to 0.1.38. The methodological quality of each retrieved review is available in the Supplementary Materials 3 (Table S4).

**Table 2:** Analysis of the temporal evolution of the methodological quality and risk of bias of the retrieved publications.

Methodological quality and risk of bias		Year of publication							
ROBIS Domain	Quality level	1999–2006		2007–2014		2015–2024			
		%	n	%	n	%	n	M (SD)	95 %CI
Study eligibility criteria	Low	11.11	1	0.00	0	0.00	0	1.38 (0.57)	0.27–2.50
	Moderate	11.11	1	8.33	2	2.22	2		
	High	77.78	7	91.67	22	97.78	88		
Identification and selection of studies	Low	11.11	1	0.00	0	2.22	2	0.85 (0.42)	−0.01-1.64
	Moderate	11.11	1	20.83	5	4.44	4		
	High	77.78	7	79.17	19	93.33	84		
Data collection and study appraisal	Low	11.11	1	8.33	2	2.22	2	1.11 (0.33)	0.47–1.76
	Moderate	44.44	4	33.33	8	12.22	11		
	High	44.44	4	58.33	14	85.56	77		
Synthesis and findings	Low	11.11	1	0.00	0	1.11	1	0.87 (0.36)	0.16–1.56
	Moderate	22.22	2	29.17	7	10.00	9		
	High	66.67	6	70.83	17	88.89	80		
Risk of bias in the review	Low	0.00	0	4.17	1	2.22	2	1.03 (0.34)	0.35–1.71
	Moderate	44.44	4	33.33	8	88.89	8		
	High	55.56	5	62.50	15	89.13	80		

Note. Data represent the percentage (%) of the quality level across the different time periods. ROBIS = the Risk of Bias in Systematic Reviews tool; n = number of publications; M = Mean of the posterior distribution for regression coefficients of Bayesian ordered regression; SD = standard deviation; 95 %CI = Bayesian credibility intervals.

### Co-occurrence network analysis

**Fig. 4.**
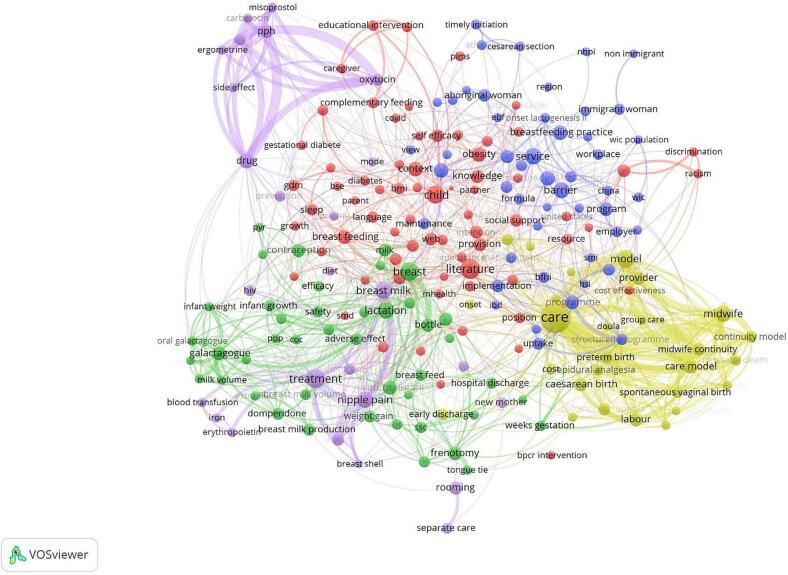
Network visualization map of terms. The node size represents the frequency of term occurrences, with the largest node and label indicating the most frequently used terms. The thickness of the connecting lines represents the frequency of co-occurrence between keywords, with the thickest links representing the most frequent co-occurrences. The different colors represent different clusters of terms. BFHI = Baby-friendly Hospital Initiative; BMI = body mass index; BSE = breastfeeding self-efficacy; COC = combined oral contraceptives; GDM = gestational diabetes mellitus; HSI = health sector initiatives; IBD = inflammatory bowel disease; NHPI = Native Hawaiians and Pacific Islanders; PIMS = perceived insufficient milk supply; POP = progestogen-only pill; PPH = postpartum hemorrhage; PVR = progesterone vaginal ring; SMI = severe mental illness; SSC = skin-to-skin contact; WIC = program for women, infants, and children.

A total of 4155 terms were used in the title and abstract fields, and 216 relevant terms that appeared in at least five retrieved reviews were included in the term co-occurrence analysis and network visualization map (Fig. 4). Cluster 1 (red), characterized by 70 terms, encompassed multiple factors including maternal mental (e.g., eating disorders and stress) and medical conditions (e.g., diabetes and obesity), sociodemographic (e.g., ethnicity and income level), and environmental factors (e.g., natural disasters and pandemic context), as well as social and cultural determinants (e.g., racism and discrimination). Cluster 2 (blue) comprised 49 terms focusing on social factors (e.g., migrant status and aboriginal minorities), workplace factors (e.g., working conditions and workplace interventions), maternal conditions (e.g., inflammatory bowel disease and autism), and health system factors (e.g., access to healthcare professionals). Cluster 3 (green) included 44 terms related to human milk features (e.g., production and composition), medical interventions (e.g., use of galactagogues and contraceptives), and infant conditions (e.g., tongue tie and preterm birth). Cluster 4 (yellow) comprised 27 terms related to the healthcare system (e.g., continuous support, midwife continuity of care models, and instrumental birth). Finally, Cluster 5 (purple) contained 26 terms primarily focused on maternal health conditions (e.g., nipple pain and postpartum hemorrhage) and their treatments and side effects (e.g., use of lanolin and erythropoietin).

### Identification of barriers and facilitators of breastfeeding practice

**Fig. 5.**
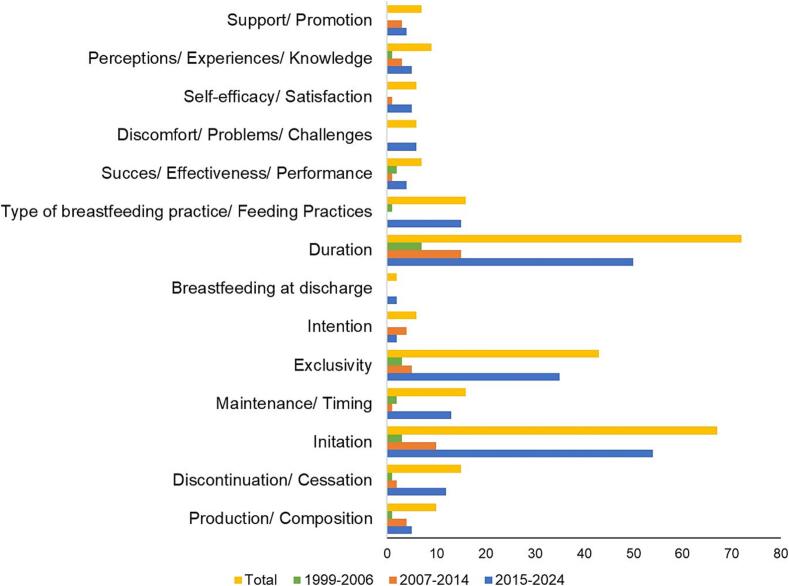
Breastfeeding outcomes assessed over time by the retrieved reviews.

The most extensively investigated breastfeeding outcomes were duration (assessed in 72 studies), initiation (assessed in 67 studies), and exclusivity (assessed in 43 studies), all of which have shown a steady increase in research focus over time (Fig. 5). Notably, recent trends indicate a growing interest in psychosocial dimensions of breastfeeding outcomes (including perceptions, experiences, and knowledge) and the support and promotion of breastfeeding. Conversely, the outcomes related to breastfeeding intention and breastfeeding at discharge remain underexplored, with only six and two studies addressing these issues, respectively.

The thematic analysis conducted on the systematically revised breastfeeding barriers and facilitators identified four key themes: Therapeutic and care interventions; Support networks and education; Maternal-Infant health issues; and Societal and Environmental Context. This analysis provides a comprehensive synthesis of the factors influencing breastfeeding, offering insights into the complex interplay between interventions, individual and community dynamics, and broader systemic and socio-cultural factors. Information about outcomes and barriers/facilitators addressed by each retrieved review is available in Supplementary Materials 3 (Table S4).

#### Therapeutic and care interventions

These interventions encompass all factors related to therapies or care interventions that impact breastfeeding outcomes for women and infants.

##### Facilitating interventions

Nipple pain, if unmanaged, can discourage mothers from continuing breastfeeding, potentially leading to early weaning and reduced breastfeeding success. Dennis et al. demonstrated that most women experience a decrease in nipple pain within the first week postpartum regardless of the treatment [31], underscoring the importance of informing mothers about this timeline to support continued breastfeeding. Preventive strategies, including proper positioning and comprehensive breastfeeding instruction, are critical for mitigating nipple pain [32]. Effective pain relief measures include warm water compresses, lanolin, and adopting a laid-back position to alleviate discomfort [33]. Also, frenotomy for treating tied tongue in infants has been found to enhance breastfeeding success by reducing pain [34].

Lactation insufficiency presents another important challenge, and interventions like domperidone-based treatments have been shown to markedly increase milk production without adverse effects on mothers or infants [35,36]. Although natural galactagogues show potential benefits, their effectiveness is not yet supported by robust evidence [37]. On the other hand, addressing postpartum anemia with erythropoietin has been associated with improved breastfeeding outcomes [38].

Birth Preparedness and Complications Readiness interventions have increased the rates of breastfeeding initiation within the first hour of life [39]. In addition, multidisciplinary approaches involving “feeding champions” and structured improvement methodologies, such as Plan-Do-Study-Act, have demonstrated nutrition benefits for newborns, including shortened hospital stays and improved weight gain [40].

Technological interventions, particularly mobile health (mHealth) applications, have been effective in promoting exclusive breastfeeding and enhancing self-efficacy, however they have little influence on early breastfeeding initiation within the first hour [41].

Skin-to-skin contact immediately after birth is beneficial for breastfeeding outcomes [[42], [43], [44]]. Similarly, vaginal delivery favors timely initiation and exclusive breastfeeding [45].

##### Hindering interventions

Conversely, certain factors constitute barriers to effective breastfeeding. Neuraxial analgesia during labor has been associated with several impacts, with some studies reporting reduced breastfeeding rates [46]. Cesarean sections are consistently linked to lower rates of breastfeeding initiation and duration [[47], [48], [49]], with specific challenges noted for women with obesity and delayed lactogenesis II [50,51]. Inadequate behavioral sleep interventions in infants during the first six months can also unintentionally lead to early cessation of breastfeeding and increased maternal anxiety [52].

Avoiding bottle feeding in favor of cup or tube feeding methods has been associated with higher rates of full breastfeeding and sustained positive outcomes [53]. Conversely, Flint et al. concluded that cup feeding did not offer clear benefits over bottle feeding for breastfeeding maintenance and may extend hospital stays [54].

##### Inconclusive or inconsequential interventions

Davie et al. reported that social-psychological interventions—including cognitive behavioral therapy, acceptance and commitment therapy, psychoanalytic and psychodynamic approaches, systemic, mindfulness, and art therapies, as well as the application of behavior change techniques—were effective in improving breastfeeding initiation rates [55]. However, these interventions did not impact overall breastfeeding duration or exclusivity, and the results were influenced by publication bias.

Breastfeeding is safe for women on opioid substitution treatment, with benefits like reduced neonatal abstinence syndrome, especially when combined with rooming-in [56]. However, stigma and inadequate education hinder breastfeeding rates, highlighting the need for supportive policies and education on the safety of opioid use during breastfeeding.

Advising increased fluid intake and using uterotonic agents for postpartum hemorrhage have shown no impact on breastfeeding outcomes [57,58]. The effects of contraceptives on breastfeeding duration remain inconsistent [[59], [60], [61]], however, Sothornwit et al. found that the immediate postpartum insertion of progestin-releasing implants and IUDs has little or no negative impact on breastfeeding [62].

Group prenatal care, involving structured programs for small groups of pregnant women, shows comparable rates of breastfeeding initiation, preterm birth, and NICU admissions to traditional single care, though it is associated with a reduced rate of low birth weight infants [63]. Although rooming-in practices seems to be associated with longer durations of exclusive breastfeeding, there is insufficient evidence to support or refute mother-infant separation versus rooming-in, suggesting the need for a well-designed randomized controlled trial to investigate the effects on breastfeeding outcomes [64]. Similarly, moderate-certainty evidence suggests that early discharge likely results in little to no difference in breastfeeding rates at both six weeks and six months compared to standard discharge, with most studies originating from high-income countries [65].

Breast reduction surgery generally does not affect breastfeeding ability, with challenges being more often attributed to psychosocial factors [66]. Other health conditions, such as maternal inflammatory bowel disease, are also compatible with breastfeeding when using certain medications [67]. Anesthetic drugs are generally safe when administered in small doses [68]. However, the effects on breastfeeding of epidural analgesia [69], hormonal contraception [70], antidepressants [71], antenatal education [72], and milk expression [73] remain inconclusive.

#### Support networks and education

This theme centers on the knowledge, skills, and support systems for women to effectively initiate and sustain breastfeeding. The crucial role of family, friends, health professionals, community, and workplace in providing support is highlighted.

##### Family and friends

Support from family and friends is essential for successful breastfeeding initiation and duration. Comprehensive prenatal interventions, as demonstrated by Wouk et al. [74], enhance breastfeeding outcomes through effective education and family involvement. Skouteris et al. emphasize that extended postpartum support, facilitated by modern communication tools, is critical in reinforcing this assistance [75]. Continuous emotional and practical support from family members improves breastfeeding outcomes, especially for adolescent mothers [76,77]. Similarly, Nelson highlights the importance of personalized support tailored to each mother's unique circumstances, which can compromise breastfeeding exclusivity and maintenance [78].

##### Healthcare professionals

Healthcare professionals play a key supporting role through education and counseling. Wouk et al. and McFadden et al. found that comprehensive prenatal education combined with peripartum support effectively improves breastfeeding initiation and duration [74,79]. The Baby-Friendly Hospital Initiative (BFHI), as noted by Beake et al. and Fallon et al. [80,81], enhances breastfeeding initiation rates. However, Schmied et al. observed challenges related to maternal autonomy and hospital resources within the BFHI [82], while Fallon et al. identified that BFHI may promote unrealistic breastfeeding expectations, fail to meet individual needs, and foster negative emotional experiences [81].

Balogun et al. demonstrated that education provided by healthcare professionals during peripartum enhances breastfeeding initiation rates [83]. By contrast, Patil et al. warn that inadequate communication and formula promotion by healthcare staff can negatively impact breastfeeding [49]. In consequence, structured support programs delivered at various postpartum stages have been shown to improve outcomes [84].

While prenatal education effectively imparts breastfeeding skills, many women face challenges in maintaining exclusive breastfeeding due to insufficient attention to maternal perceptions of infant behavior and unresolved concerns about milk supply [85]. Pregnant women need early and realistic information about breastfeeding with long-term support, emphasizing breastfeeding as a personal choice rather than a social obligation, addressing specific emotions such as embarrassment and guilt, and fostering positive self-views to empower them and enhance breastfeeding behaviors [86].

##### Community

Multidisciplinary approaches and global perspectives highlight the importance of community-based support and culturally tailored interventions. Doerzbacher and Chang found that strategies emphasizing maternal-infant bonding, such as midwife-led continuity models, optimize breastfeeding outcomes [87]. Community-based interventions, including groups of women and home-based care, are cost-effective to increase breastfeeding rates [88]. Additionally, interventions grounded in the self-efficacy theory and those integrating both professional and layperson support have shown positive effects [89,90], with current evidence suggesting that tailored home-visiting schedules could further enhance exclusive breastfeeding rates [91]. Continuous support from a trained laywoman (doula) during childbirth improves obstetrical and postpartum outcomes, with higher rates of breastfeeding initiation and exclusive breastfeeding at six weeks, including a notable increase among doula-supported teens compared to the USA average [92].

The effectiveness of continuous support during labor on breastfeeding outcomes needs to be clarified. Bohren et al. reported that its impact on breastfeeding is inconclusive [93]. Sandall et al. observed that midwife-led continuity models do not affect breastfeeding initiation rates, although they offer other benefits, such as reduced cesarean section rates [94]. Meedya et al. identified modifiable factors that influence breastfeeding decisions, such as breastfeeding intention, self-efficacy, and social support, noting that current midwifery promotion strategies often focus on social support but insufficiently address the modification of breastfeeding intention and self-efficacy [95].

Various initiatives have been proposed to protect and promote breastfeeding as part of community-based support. Seward et al. found that breastfeeding support groups enhance the likelihood of initiating breastfeeding within the first hour after birth in rural settings [96]. Social media support groups, as highlighted by Morse and Brown [97], promote knowledge, social connections, and confidence among mothers, improving their breastfeeding experiences. Hunt et al. and Fairbank et al. found that integrating peer support with health services notably improves breastfeeding outcomes, especially in disadvantaged groups [98,99]. Fairbank et al. specifically highlighted the effectiveness of antenatal education and peer support in increasing breastfeeding initiation and duration across diverse socioeconomic contexts [98].

Supportive education and counseling programs are key strategies. Gavine et al. found that providing women with extra organized support reduces the risk of stopping breastfeeding before six months and promotes exclusivity [100]. Limited breastfeeding knowledge, delayed initiation, and formula use are key factors contributing to perceived insufficient milk supply, which affects about 50 % of women who discontinue breastfeeding [101]. Wong et al. emphasize that educational and support interventions delivered both prenatally and postnatally increase exclusive breastfeeding rates [102]. Lavender et al. found that telephone support might increase breastfeeding duration, though results were inconsistent [103], indicating a need for further research. Educational interventions, as concluded by Arikpo et al. [104], favor breastfeeding, reduce the early introduction of semisolid foods, and enhance caregiver hygiene practices.

##### Tailored support

Although support networks and education may positively influence breastfeeding outcomes, certain groups of women require special attention. Regarding culturally tailored support, insufficient knowledge and cultural biases, such as a preference for formula feeding, contribute to lower breastfeeding rates in Saudi Arabia [105]. African American women also benefit from targeted interventions [106]. In Canada, immigrant mothers face challenges in initiating and sustaining breastfeeding due to inadequate culturally relevant support [107]. Furthermore, culturally supported practices such as bed-sharing have increased both the frequency and duration of breastfeeding [108]. Lumbiganon et al. advocate for more comprehensive research on antenatal breastfeeding education in low- and middle-income countries to address these diverse needs [109].

Regarding health conditions, HIV-positive women in Africa face barriers such as inadequate support and insufficient knowledge about viral transmission, which hinder exclusive breastfeeding despite high initiation rates [110]. Women with obesity benefit from support provided by both family and healthcare professionals to enhance breastfeeding success [50]. Da Silva Tanganhito et al. emphasize that appropriate training for healthcare professionals is essential for supporting breastfeeding among women with postnatal depression, alongside mental health interventions [111]. According to Whitford et al. [112], there is a gap in targeted breastfeeding education and support for women with twins or higher order multiples, given that none of the studies reviewed by the authors provided specialized training or tailored support, and no randomized controlled trials assessed the most effective forms, timing, or providers of such support.

##### Workplace

Workplace policies must be addressed. Vilar-Compte et al. reported that interventions such as lactation rooms and flexible work arrangements are essential for protecting breastfeeding [113]. Moreover, workplace policies providing lactation rooms and breastfeeding breaks enhance initiation rates and exclusive breastfeeding [114]. Dinour and Szaro also highlight the positive impact of employer-based support [115]. Similarly, Hirani and Karmaliani confirm the importance of maternal education to manage breastfeeding during work and employer awareness about its benefits [116]. For instance, policies promoting skin-to-skin contact immediately after delivery are positively associated with breastfeeding outcomes in military women [117].

#### Maternal-infant health issues

This theme encompasses a broad range of physical and psychological conditions and related social factors that impair the health and well-being of mothers and infants. These conditions can greatly influence various aspects of breastfeeding.

##### Maternal issues

Maternal obesity and overweight are significant barriers that reduce breastfeeding initiation and maintenance [118]. Obesity increases the risk of early breastfeeding cessation, with many women reporting insufficient milk as a reason for stopping breastfeeding. These women frequently experience physiological challenges, such as low milk production with delayed lactogenesis II and difficulties with positioning due to a larger breast size [50]. García et al. noted a 1.11-fold increase in the relative risk of breastfeeding cessation per body mass index category [119]. Amir and Donath reported that women with obesity breastfed for shorter periods even after adjusting for confounding factors [120]. Although Fair et al. point out a lack of evidence on the effectiveness of interventions for promoting breastfeeding in overweight or obesity [121], Reichental et al. demonstrate that targeted interventions in obesity and gestational diabetes improved breastfeeding outcomes [122].

Diabetes, including gestational and type 2 diabetes, exerts substantial barriers to breastfeeding. Taylor et al. reported that infants of mothers with diabetes often face complications such as prematurity, macrosomia, and cesarean delivery, which can hinder breastfeeding initiation. However, breastfeeding challenges are less pronounced when gestational diabetes is controlled [123]. In this sense, infants exposed to gestational diabetes are approximately 40 % more likely to receive formula or supplementary milk before hospital discharge and 30 % less likely to continue breastfeeding after 12 months [124]. Also, 31 % of women with diabetes present delayed lactogenesis II onset, showing risk factors such as maternal age over 35 years, primiparity, maternal overweight/obesity, cesarean section, anxiety, depression, and gestational hypertension [51].

Psychological factors also play a key role in breastfeeding practices. Lyons et al. identified key barriers, including intentions to breastfeed, belief in human milk's nutritional adequacy, body image concerns, and social support knowledge [125]. Women with obesity often have a negative body image, face social stigma, and have low confidence in their ability to breastfeed, which further impairs breastfeeding [50]. On the other hand, Badr and Zauszniewski found that maternal postpartum fatigue has a medium to large effect on breastfeeding difficulties and is correlated with stress, anxiety, and depression [126]. Indeed, postpartum depression exacerbates these challenges. Butler et al. reported a negative association between perinatal depressive symptoms and breastfeeding exclusivity and maintenance [127]. Similarly, Dennis and McQueen reported increased breastfeeding difficulties and lower breastfeeding self-efficacy among women with depressive symptoms [128]. Da Silva Tanganhito et al. noted the impact of physical pain and fatigue on women with postpartum depression, leading to breastfeeding difficulties in the absence of integrative healthcare support [111]. Oyetunji and Chandra confirmed this situation by reporting that postpartum stress and depression disrupt breastfeeding, which impairs infant development (e.g., language development, cognitive skills, motor skills, and sleep patterns) [129].

Severe mental disorders represent additional challenges. Baker et al. noted that affected women are less likely to initiate and continue breastfeeding, with inconsistent advice from healthcare professionals and insufficient support [130]. De Jager et al. emphasize that psychosocial factors such as self-efficacy, postpartum depression, and maternal breastfeeding intentions strongly predict exclusive breastfeeding outcomes [131]. Other factors, such as anxiety and social support, also determine breastfeeding duration. In the same vein, eating disorders (EDs) affect breastfeeding. Kimmel et al. found that women with anorexia nervosa are more likely to cease breastfeeding before six months, although initiation rates are similar across different EDs [132]. Kaß et al. reported mixed results regarding breastfeeding duration, with larger studies suggesting a shorter duration for women with EDs, particularly anorexia nervosa [133]. Emotional problems associated with EDs profoundly impact both the maternal-child feeding environment and breastfeeding success. Similarly, Grant et al. noted that autistic mothers often face sensory challenges and inadequate support, affecting their breastfeeding experiences [134].

Among infectious diseases, reviews mainly address HIV and congenital Zika syndrome. John et al. estimated a 16 % risk of HIV transmission through human milk, with transmission rates varying based on breastfeeding duration [135]. However, this evidence should be interpreted in the current context. In this sense, despite WHO guidelines recommending exclusive breastfeeding for six months in low-income settings, HIV-positive women face cultural and practical barriers that contribute to early discontinuation of exclusive breastfeeding [110]. Congenital Zika syndrome results in feeding difficulties, with a high prevalence of dysphagia among affected infants [136].

Women with breast cancer are also challenged. Bhurosy et al. found that while some women benefit from support and motivation to breastfeed, others encounter obstacles such as medical advice against breastfeeding, fatigue, and reliance on a single breast [137]. Similarly, women with inflammatory bowel disease may experience unique challenges linked with their pharmacological treatment, though evidence suggests that breastfeeding does not increase the risk of disease flare-ups and might have protective effects against relapse [67]. On the other hand, Gray et al. emphasize the role of modifiable factors like diet and substance use in changing breastfeeding outcomes [138]. Additionally, short birth intervals between pregnancies can hinder successful breastfeeding due to physiological regression and maternal fatigue [139].

##### Infant issues

Infant anatomical issues, such as tongue tie, substantially impact breastfeeding. O'Shea et al. and Bruney et al. highlight that tongue-tie and associated nipple pain can impede feeding, with frenotomy often improving these difficulties [34,140].

#### Societal and environmental context

This theme focuses on how population characteristics and contexts determine breastfeeding outcomes.

##### Origin-related disparities

Dalili et al. revealed regional differences in breastfeeding duration in Iran [141]. In this sense, women living in regions with effective family health programs reported the longest duration of breastfeeding. Furthermore, longer durations were positively associated with older maternal age, lower education levels, higher birth order, family support, nighttime breastfeeding, rural residence, and planned pregnancies. Conversely, authors found that early breastfeeding cessation was more common among women with higher education levels and employment. Interestingly, income, healthcare access, underlying diseases, maternal body mass index, and childbirth experience did not influence breastfeeding duration within this context.

Adams et al. reported that middle-income mothers breastfeed more frequently than low-income mothers, observing variations across ethnic groups [142]. Factors such as being white, older, married, educated, and having the intention to breastfeed were associated with higher breastfeeding rates. Indeed, Hedberg identified that non-Hispanic ethnicity, combined with maternal health issues (e.g., obesity and depression) and demographics (e.g., younger age and incomplete high school education), is associated with lower breastfeeding rates [143].

Robinson et al. reported that racism and biased assumptions from healthcare providers are obstacles to breastfeeding among African American women in the USA, resulting in fewer referrals for lactation support and limited assistance [144]. This contributes to persistent disparities in breastfeeding rates among ethnic minority women in this country [145]. Furthermore, Johnson et al. highlight the lack of targeted interventions for African American women, although institutional strategies, such as lactation consultants and structured prenatal care, could overcome this situation [146]. Interpersonal support from peers and family is also crucial in influencing breastfeeding behaviors among low-income African American women, underscoring the importance of social support networks [106].

Regarding native populations, such as Aboriginal and Torres Strait Islander communities from Australia, Springall et al. identified several protective factors of breastfeeding, including residing in remote areas, accessing aboriginal-specific services, higher education levels, and increased maternal age. However, various risk factors, such as smoking during pregnancy and admission to specialized care units, were adverse to breastfeeding outcomes [147]. Breastfeeding initiation rates in these Aboriginal communities are generally high at 78 %; however, exclusive breastfeeding at six months frequently falls short of Australian and WHO standards [148]. Additionally, Mitchell et al. highlight cultural practices, the normalization of bottle feeding, and the stigma associated with public breastfeeding as important factors influencing breastfeeding practices among these women [149].

Immigrant women also face challenges and opportunities in breastfeeding. Higginbottom et al. reported their barriers to accessing maternity care services, including lack of information, inadequate support, and discordant expectations between women and healthcare providers [107]. Nonetheless, Dennis et al. found that immigrant women in Canada are more likely to initiate and continue breastfeeding compared to non-immigrants, though exclusivity remains a challenge for both groups [150].

##### Health-related disparities

Societal stigma related to specific health conditions is another prominent determinant. Chang et al. identified that feeling embarrassed about breastfeeding in public and the stigma associated with obesity are relevant barriers for some women [50]. These cultural attitudes contribute to social discomfort and inhibit breastfeeding by fostering environments where public breastfeeding is viewed negatively, affecting maternal confidence and reducing breastfeeding initiation and continuation. Similarly, Grant et al. highlight that societal stigma and inadequate social support affect autistic mothers, with cultural perceptions surrounding autism exacerbating their difficulties in breastfeeding [134]. Also, Vitalis et al. confirm that exclusive breastfeeding duration remained brief due to HIV transmission concerns, work obligations, and cultural factors, despite high initiation rates [110].

##### Environmental context

Environmental factors, such as natural disasters and pandemics, further exacerbate breastfeeding challenges. Ratnayake Mudiyanselage et al. highlighted as facilitators the availability of privacy, community support, and adaptation of professional support to local conditions, whereas decreased self-efficacy and limited resources pose considerable barriers [151]. Similarly, Adesanya et al. examined the impact of the COVID-19 pandemic on breastfeeding, noting that healthcare providers struggle to offer consistent care due to knowledge gaps and limitations of virtual healthcare [152].

##### Legislative context

Federal and state laws have a meaningful impact on breastfeeding initiation and duration, particularly benefiting minority women [153]. This legislative context, for example, mandates employers to provide breaks and lactation spaces, which promote the practice among Hispanic women and its initiation among African American women in the USA. This can be potentiated by the BFHI and comprehensive maternity care interventions.

As a particular case, women in the military require tailored policies, as Owens and Di Tomasso explored, including key factors such as hospital practices, maternity leave duration, and workplace support [117]. Notably, perinatal policies promoting early skin-to-skin contact improve breastfeeding initiation and duration among these women serving in the USA, facilitating early bonding and successful breastfeeding within the particularly structured military environment.

## Discussion

To the best of our knowledge, this is the first overview of reviews to synthesize the evidence on breastfeeding barriers and facilitators, providing a comprehensive overview of this complex phenomenon. We identified 123 eligible systematic reviews, with the majority being of good methodological quality and published in high-impact journals. We observed notable trends over time, including increased attention to psychosocial and cultural determinants and outcomes, increased representation of studies from low- and middle-income countries, a rise in the use of qualitative systematic reviews, and an improvement of methodological quality. Despite these enhancements, the geographical representation remains skewed towards high-income countries, and some breastfeeding outcomes require further exploration. Through thematic analysis, we identified several determinants related to breastfeeding outcomes grouped in major dimensions.

Our bibliometric analysis provides a baseline of publication trends and the impact of reviews on breastfeeding determinants. The number of reviews has increased over the last 25 years, with 73 % of the reviews published in the most recent period (2015–2024). The majority of reviews had corresponding authors affiliated with institutions from high-income countries. This aligns with the study by Andersen et al. [154], who found that the publication of systematic reviews is rapidly increasing. Compared to other general publications in the health sciences that have a growth rate of 5 %, systematic reviews are growing at an average annual rate of 26 %. Additionally, the authors observed that English-speaking and high-income countries produce the majority of these studies.

The increase in systematic reviews of breastfeeding is influenced by several factors. More midwives and allied health professionals are pursuing advanced degrees to enhance their research skills [155,156]. This educational growth encourages practitioners to participate in investigations and evidence synthesis to address knowledge gaps [157]. Furthermore, the shift towards an evidence-based healthcare model requires reliable data to support clinical practice [158]. Collaboration between healthcare providers and researchers facilitates a timely movement of the best available evidence into practice [159], with robust systematic reviews increasingly being published as essential tools.

### Methodological approach of scientific evidence

Regarding breastfeeding literature, Sabancı Baransel et al. observed that research on breastfeeding developed slowly until the 2000s, after which it saw a notable acceleration in growth [160]. Consistent with our findings, the USA emerged as the leading country in terms of breastfeeding research output. Furthermore, these authors noted that the psychological aspects of breastfeeding have been intensely debated, particularly in recent years, in alignment with our current study. Similarly, another study revealed an increase over time in the annual number of meta-analyses and systematic reviews related to breastfeeding and human milk. This increase was expected, considering that such methods were almost nonexistent before the 1990s and required a substantial cumulative sample size along with an adequate number of primary studies [161]. Additionally, the number of published clinical trials related to breastfeeding have tripled over time, which likely contributed to the growth of systematic reviews and meta-analyses in this field.

In the current overview of reviews, the Cochrane Database of Systematic Reviews was the most active journal publishing reviews on breastfeeding determinants and had the greatest impact in terms of citations. This may be attributed to the generally higher quality of reviews published in Cochrane, which are highly valuable for the development of health policies [154]. Additionally, these articles have surpassed 100 citations, a benchmark commonly used to designate a work as a “classic” [162]. While older publications generally accumulate more citations than recent ones, regardless of their impact [163], our findings indicate that citation density has increased in recent years. It is important to emphasize that although the number of citations can reflect an article's influence within a specific research field, it does not necessarily equate to its scientific value [164].

The publication of meta-analyses has increased, likely driven by the growing number of primary studies necessary for their development. Meta-ethnographies have gained prominence due to their systematic approach to synthesizing qualitative research that provides profound insights into complex issues. In addition, it is supported by the advancement of methodological frameworks and the proliferation of specialized journals [165,166]. This method focuses exclusively on qualitative data from the social sciences and utilizes original interpretations of primary studies, which undergo analytical synthesis that facilitates knowledge transfer to healthcare [167]. A comparable growing trend was found for mixed-method systematic reviews [166]. These reviews combine quantitative and qualitative evidence into a single review, integrating diverse types of data to deliver a more thorough understanding of complex phenomena [168]. By utilizing both statistical information and contextual insights, they offer a deeper, more nuanced perspective that can enhance interdisciplinarity in decision-making and policy development across various fields [169].

### Social ecological model of breastfeeding

Current evidence on the barriers and facilitators of breastfeeding predominantly originates from high-income countries with an extensive history of research in breastfeeding, although participation from middle- and low-income countries has increased in recent years [160]. The identified themes can be interpreted within the framework of the Social Ecological Model of Health (SEM), a comprehensive conceptual model that highlights the dynamic interplay between individuals and their environment, emphasizing the influences of individual, interpersonal, community, organizational, and policy-level factors on health-related decisions [170,171].

The individual level encompasses a range of intrapersonal biological and psychological characteristics [172]. Extensive research on breastfeeding has explored this. For instance, the theme “Maternal-infant health issues” illustrates how various health issues (e.g., maternal obesity [50,[118], [119], [120]], diabetes [51,123,124], mental illness [111,126,[128], [129], [130], [131], [132], [133], [134]], and anatomical problems in infants [34,140]) affect breastfeeding. The theme “Support networks and education” emphasizes the pivotal role of maternal knowledge and skills. Also, the evidence highlights that comprehensive perinatal education is crucial, as it provides mothers with the necessary information and skills for coping with breastfeeding [74,79,83,100,102,104,116]. The theme “Therapeutic and care interventions” underscores that effective management of nipple pain [32,34] and lactation insufficiency [35,36], and neonatal practices (e.g., immediate skin-to-skin contact and rooming-in [43,44,56,64]) enhance breastfeeding success. Conversely, cesarean section and neuraxial analgesia negatively impact breastfeeding initiation and duration [[46], [47], [48], [49]]. Other interventions show inconclusive or minimal effects [[59], [60], [61], [62]]. Overall, this overview of reviews provides a thorough understanding of individual-level factors combined with tailored and evidence-based interventions.

The interpersonal level of the SEM underscores the importance of both formal and informal support systems [173,174]. The theme “Support networks and education” reveals that effective support is pivotal for successful breastfeeding, encompassing emotional, practical, and logistical assistance from family [76,77], healthcare professionals [74,79,87,88], and social networks [75,96,97,99]. Prenatal interventions that actively involve family members enhance breastfeeding initiation and duration [77], while extended postpartum support, often facilitated through modern communication tools [75], helps maintain breastfeeding efforts. Support groups, especially in rural settings, favor early breastfeeding initiation [96,97,99]. Tailored support is critical [78], particularly for overcoming challenges faced by specific populations, including HIV-positive women [110], those with obesity [50] and mental health issues [111], and adolescent mothers [76,77]. Additionally, culturally relevant interventions are essential for helping racialized ethnic groups and immigrant women [106,107]. In consequence, a well-rounded support system is fundamental for optimizing breastfeeding outcomes and addressing diverse needs.

The organizational and community levels are closely interconnected and explore how institutions, social networks, and community resources influence health outcomes and related behaviors [173,174]. The theme “Support networks and education” underscores the pivotal role that healthcare infrastructure and regulations play in influencing breastfeeding. Effective healthcare systems, encompassing hospital protocols, midwifery practices, and community-based care, are key in supporting breastfeeding [39,87,88]. Comprehensive perinatal education programs enhance breastfeeding initiation and duration, with support interventions being also beneficial, especially for disadvantaged groups [87,89,90,99]. Workplace policies that include lactation rooms and flexible work arrangements are critical for maintaining breastfeeding [[113], [114], [115], [116]]. Training for healthcare providers ensures consistent evidence-based support [48,111], although inconsistent communication and formula promotion can impair breastfeeding success [48]. These findings highlight the need for integrated, well-structured policies and programs that should undergo high-quality evaluation to address the diverse needs of mothers and infants, ultimately fostering a supportive environment [175].

The last level of the SEM encompasses broad social and cultural norms, policies, and context [176]. Additionally, the economic and political context, mass media influence, and institutional practices are part of this level [153]. The theme “Societal and environmental context” shows that social norms and cultural beliefs influence attitudes towards breastfeeding, shaping both individual behavior and community support. For example, societal stigma and embarrassment associated with public breastfeeding can deter mothers from initiating or continuing breastfeeding [50,149]. Discrimination and racism also play a critical role, leading to biased assumptions from healthcare providers, reduced support, and lower breastfeeding practice [[143], [144], [145], [146]]. Additionally, societal attitudes towards specific health conditions, such as obesity and autism, contribute to stigma [50,134]. Conversely, supportive policies and legislation that mandate workplace accommodations for breastfeeding address its socio-cultural barriers [117,153]. On the other hand, countries show a differential responsiveness to breastfeeding promotion interventions, such as peer support policies, with low- and middle-income ones being more sensitive, whereas high-income countries already include breastfeeding support as part of routine postnatal healthcare [177]. Moreover, women from high-income settings often face fewer barriers to breastfeeding due to adequate support and resources than other ones, with low- and middle-income groups requiring further interventions [39,93,142].

Regional disparities also exist, with better family health coverage in economically stable areas being linked to better breastfeeding outcomes [141]. Thus, this level shapes the breastfeeding experience, underscoring the need for culturally sensitive interventions.

**Fig. 6.**
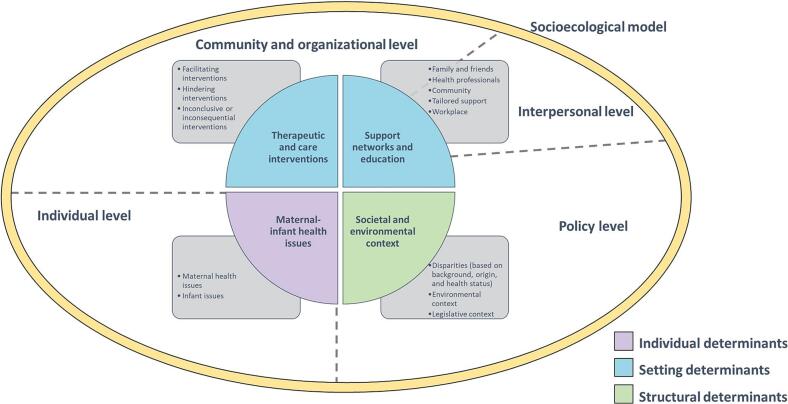
Themes identified in this overview of reviews regarding breastfeeding facilitators and barriers, mapped onto the Socioecological Model of Health and the Conceptual Model of an Enabling Environment for Breastfeeding.

The themes identified in this study also align with the conceptual model of an enabling environment for breastfeeding proposed by the Lancet Breastfeeding Series Group [10]. This model classifies the determinants influencing breastfeeding decisions and practices into three levels: 1) individual level, including maternal and infant attributes, as well as the mother-infant relationship; 2) setting level, including health systems and services, family and community, and workplace and employment; 3) structural level, including broader influences such as sociocultural and market context. The model emphasizes the intricate interplay of these factors shaping breastfeeding over time and serves as a framework for implementing comprehensive strategies to address multilevel barriers effectively. These strategies encompass interventions such as social mobilization and mass media campaigns; legislation, policy development, financing, monitoring, and enforcement; and enhanced counseling, support, and lactation management services. Fig. 6 illustrates the themes identified in this study, contextualized within the SEM and the determinants of an enabling environment for breastfeeding.

### Implications for healthcare practice and research

In this comprehensive review, we synthesized the available evidence on the barriers and facilitators of breastfeeding, providing a detailed analysis of current research trends. The findings reveal that factors at various levels influence breastfeeding outcomes, which has significant implications for healthcare and maternal-child health policies. Specifically, vulnerable groups were identified, including women with physical or psychological conditions, ethnic minorities, those from low-income backgrounds, and working mothers, who require focused professional and social support. Additionally, there is a need to restructure the healthcare system to provide equitable, fair, and comprehensive care with well-trained professionals. Research has shown that interdisciplinary approaches to maternal care and strategies such as the BFHI are strongly associated with positive breastfeeding outcomes. It is also crucial to review breastfeeding protection policies, especially those related to employment, as workplace policies supporting breastfeeding demonstrate notable benefits. Addressing social stigmas associated with breastfeeding in neurodiversity and other conditions is also imperative. The results also offer insights for emergency and crisis situations, although these contexts warrant further exploration. Despite the growing attention to social factors, more studies are needed to explore the challenges faced by specific populations, such as LGBTQ+ minorities, ethnic groups, and religious communities. Finally, while research on breastfeeding barriers and facilitators is increasing, the evidence largely comes from high- and middle-income countries. Future research should focus on the determinants of breastfeeding in low-income countries.

Understanding the local cultural context allows the identification of subtle but highly influential factors that can facilitate or hinder breastfeeding. Concerning this, mothers from Ghana perceive that grandmothers did not practice exclusive breastfeeding but children grew well, gestures of babies suggested their readiness to start eating, and human milk was watery and did not satisfy or nourish infants. Moreover, they provide corn flour mixed with water or light porridge during the first few days after birth to welcome newborns [178]. Meanwhile, during the first six months, breastfeeding practices in Nigeria varied from exclusivity to mixed feeding due to traditional practices centered on the symbolic, nutritional, and religious roles of water [179]. In Kenya, some beliefs result in suboptimal breastfeeding practices, such as considering colostrum as curdled milk, fear of being cursed associated with breastfeeding (while engaging in extramarital affairs or being practiced in public), and sagging breasts [180]. Outside Africa, perceptions of insufficient milk supply, infant illnesses, and breast problems also limit exclusive breastfeeding in Indonesia [181]. In Spain, midwives recognize poor breastfeeding culture based on gender inequities, negative messages about its practice, artificial feeding as a norm, and the belief that breastfeeding is incompatible with social life and recreational activities. These factors currently converge into a social construct of motherhood that can negatively affect exclusive breastfeeding [182].

The ideology and sentiment of breastfeeding are sociohistorical constructions. Currently, the decision to breastfeed in African American women is shaped by the generational trauma of wet nursing during slavery. This practice is inherently linked to white supremacy, medical racism, and the physical, emotional, and mental abuse that enslaved women endured [183]. Thus, the findings highlight the need for culturally appropriate counseling services for breastfeeding, not only for mothers but also for their families and communities. Additionally, breastfeeding promotion strategies should focus on enhancing knowledge and problem-solving skills by considering individual and social contexts.

Another relevant aspect is the mobilization of foreign cultural elements to other geographical locations through immigration and colonization. For instance, Phonyiam and Berry underscored the importance of culturally tailored interventions to support breastfeeding among Asian immigrants and refugee women in the USA [184]. Postpartum care practices in Asian cultures, such as Cambodian, Chinese, Korean, and Vietnamese traditions, emphasize culturally specific diets, rest, and family support to enhance recovery and breastfeeding. Cambodian women associate breastfeeding with cultural identity, although some traditional practices are adapted in the USA. Chinese Zuo Yuezi and Korean San Hu Jo Ri highlight confinement, specialized diets, and elderly guidance, with mixed outcomes on recovery and milk production. Vietnamese care integrates balanced “hot” and “cold” diets to support maternal and infant health. Across these communities, the role of grandparents, diet, and culturally specific postpartum care practices significantly shaped breastfeeding outcomes [184]. Conversely, British colonization has meant dispossession of land and limited access to culturally safe healthcare, malnutrition, and loss of language through residential schools, loss of culture and traditional knowledge through assimilation and separation of families, disruption of breastfeeding practices, and limiting income for infant formula in different indigenous populations from ex-colony countries [185].

In Table 3, we present ten recommendations for health care and research in breastfeeding based on the reviews.

**Table 3:** Synthesis of the recommendations for healthcare and research on breastfeeding.

1. **Enhance Comprehensive Breastfeeding Support:** Schedule continuous visits with trained professionals and recommend doula support during labor. Develop targeted interventions and counseling for mothers with health issues such as diabetes and obesity, and ensure that healthcare staff are well-trained to address these needs. 2. **Promote Immediate Postpartum Practices:** Encourage early skin-to-skin contact immediately after delivery, including post-Cesarean sections. Develop and evaluate focused breastfeeding guidelines for vulnerable groups to improve breastfeeding initiation and maternal and infant outcomes. 3. **Develop and Assess Culturally Sensitive Interventions:** Create and implement educational programs involving family and traditional birth attendants. Conduct research to evaluate the effectiveness of these programs and address the impact of racism, ethnophobia, and discrimination on breastfeeding in diverse populations. 4. **Integrate and Improve Healthcare Support Systems:** Enhance collaboration between peer support and professional care for breastfeeding mothers. Address challenges faced by women with breast reduction surgery or breast cancer, and improve mental health training for healthcare professionals to support comprehensive maternal mental health. 5. **Foster Family and Community Involvement:** Engage family members and community leaders in breastfeeding support initiatives. Establish evidence-based guidelines tailored to various cultural and socioeconomic contexts, and improve policy coordination and sensitivity among healthcare professionals regarding the importance of breastfeeding. 6. **Conduct High-Quality Clinical Trials Breastfeeding Research:** Continue high-quality randomized controlled trials to evaluate the effectiveness and safety of breastfeeding interventions, including treatments for issues like nipple pain and milk insufficiency. Study large cohorts to understand the impact of physical and mental health issues on breastfeeding and infant development. 7. **Investigate Determinants of Breastfeeding and Amplify Women's Voices:** Utilize qualitative and mixed-method research to gain insights into the factors influencing women's infant feeding decisions and behaviors in different contexts and cultures. Conduct longitudinal studies to identify key determinants breastfeeding, and use these findings to develop and refine targeted interventions. Ensure that the perspectives and experiences of diverse women are included and highlighted in the research process to inform and enhance breastfeeding support strategies. 8. **Support Culturally Safe Care and Policy Implementation:** Assess the impact of educational interventions and culturally safe care for underserved populations, including Indigenous women. Scale up successful models like the Baby Friendly Initiative to diverse settings and ensure culturally sensitive staff training. 9. **Explore Maternal Mental Health and Workplace Support:** Investigate the relationship between maternal mental health and breastfeeding outcomes, focusing on postpartum depression and neurodiversity. Study the effectiveness of workplace lactation interventions and support programs for working mothers to enhance breastfeeding continuation, especially in low-income settings. 10. **Address Breastfeeding in Emergencies and Natural Disasters:** Develop and implement strategies to support breastfeeding during emergencies and natural disasters. Ensure that emergency response plans include provisions for maintaining breastfeeding practices, including the distribution of breastfeeding supplies and access to trained lactation support, and conduct research to evaluate the effectiveness of these strategies in crisis situations.

### Strengths and limitations of the study

Finally, while this overview of reviews aimed to provide a comprehensive examination of breastfeeding barriers and facilitators, several limitations may impact the robustness of the findings. Many of the included reviews featured quasi-experimental and observational studies, indicating that the determinants identified may be more accurately described as correlates rather than direct causes. Despite these limitations, the overview of reviews offers an extensive overview of the current literature, encompassing over 120 reviews and 2941 primary articles, employing a rigorous methodological framework in line with JBI guidelines and a publicly available protocol. The inclusion of qualitative reviews enhances the study by capturing the perspectives of women, family members, and healthcare professionals, thereby offering valuable insights for improving healthcare and research.

The review may not fully reflect the most recent advancements in the field, as new primary studies may have been published since the most recent bibliographic search [186]. The Web of Science was not included in the search due to accessibility limitations, which may have limited the comprehensiveness of the bibliographic search. However, this study followed the guidelines for optimal database combinations in overviews of reviews as provided by Goossen et al. [187], who identified MEDLINE as the main source of systematic reviews. The combination of MEDLINE and manual reference checking, as performed in our study, was found to retrieve over 93 % of health-related reviews, rising to 99 % when combined with additional databases. Furthermore, only peer-reviewed academic literature was considered, which excluded a significant body of gray literature that could potentially provide additional insights into breastfeeding barriers and facilitators. Variability in outcome definitions among studies and the focus on systematic reviews might have overlooked individual studies with robust evidence that has yet to be synthesized. Although the overall quality of the systematic reviews included was high, the variability in methodologies and terminologies, coupled with the heterogeneous quality of their primary studies, suggests that the findings should be interpreted with caution. Furthermore, most reviews lacked information on publication bias, which may further limit the interpretation of findings. Finally, a certainty assessment could not be performed due to the lack of a standardized procedure for conducting overviews of reviews [188].

This overview of reviews incorporates a scientometric analysis to offer a comprehensive overview of research on breastfeeding determinants, identifying key dimensions of barriers and facilitators through term network clustering. This approach provides an objective and detailed view of the field's current state and its impact [186]. Trends were quantitatively assessed using a comprehensive bibliographic dataset. However, it is important to note that the evaluation of the impact of the included reviews was based solely on citation counts for Web of Science. This platform does not provide citation information for non-indexed gray literature, unlike platforms such as Google Scholar [189].

On the other hand, this overview of reviews primarily employed a qualitative approach, using thematic analysis to identify and synthesize the main barriers and facilitators across the included studies. Given the qualitative nature of the method, our focus was on understanding the underlying themes rather than quantifying associations between variables. Thus, due to the nature of this study and the data reported in the reviewed publications, many of which are qualitative studies or studies not directly related to clinical research, effect size estimates were not consistently reported across studies. This limited our ability to present such measures uniformly. One limitation of this approach is that thematic analysis can introduce some subjectivity in interpreting themes, especially when the studies included varied in design, context, and reporting. Despite these challenges, we used rigorous methods to ensure a comprehensive and balanced identification of key themes, taking care to maintain consistency in theme extraction across studies. In future research, we recommend that studies report effect sizes, as outlined in the recommendations [190], alongside qualitative insights to enable a more nuanced interpretation of both clinical significance and thematic relevance.

Additionally, potential overlap in primary study results across reviews was not examined in detail due to resource constraints, and the diversity of methodologies used in the reviews complicated the comparison and interpretation of results, particularly in intervention studies. Given that only 40 % of the reviews evaluated for methodological quality by two authors, potential biases may have been introduced; however, pre-established criteria, methodological training, and pilot testing were employed to mitigate these biases, and inter-rater agreement was further confirmed [191]. In this context, this overview of reviews serves as a comprehensive synthesis of existing research on breastfeeding barriers and facilitators, highlighting potential paths for future research. However, it should not be interpreted as definitive evidence of causal relationships among the identified determinants.

## Conclusion

In summary, this overview of reviews provides a thorough synthesis of existing evidence on the barriers and facilitators of breastfeeding, revealing several key results. Notably, it identifies that psychosocial and cultural factors, healthcare interventions, and policies significantly impact breastfeeding outcomes. The review highlights increased attention to psychological and social determinants and a broader representation of studies from low- and middle-income countries. However, it also underscores that the majority of evidence comes from high-income countries, and some breastfeeding outcomes remain underexplored. The review points to the need for focused support for vulnerable groups, including women with physical or psychological conditions, ethnic minorities, and low-income or working women. It also emphasizes the importance of restructuring healthcare systems for equitable care, enhancing workplace policies to support breastfeeding, and addressing social stigmas. These findings call for further research in low-income countries and among specific populations.

## Funding statement

Funding for this study was provided by the Secretaría de Ciencia y Tecnología, Universidad Nacional de Córdoba (grant number: RESOL-2023-258-*E*-UNC-SECYT#ACTIP; https://www.unc.edu.ar/ciencia-y-tecnolog%C3%ADa), with EAS as the recipient. The funder was not involved in the study design, data collection or analysis, decision to publish, or manuscript preparation.

## CRediT authorship contribution statement

**Agustín Ramiro Miranda:** Writing – review & editing, Writing – original draft, Visualization, Validation, Project administration, Methodology, Investigation, Formal analysis, Data curation, Conceptualization. **Paula Eugenia Barral:** Writing – review & editing, Validation, Methodology, Investigation, Formal analysis, Data curation. **Ana Veronica Scotta:** Writing – review & editing, Writing – original draft, Validation, Methodology, Investigation, Formal analysis, Data curation. **Mariela Valentina Cortez:** Writing – review & editing, Writing – original draft, Validation, Investigation, Formal analysis. **Elio Andrés Soria:** Writing – review & editing, Writing – original draft, Validation, Resources, Project administration, Investigation, Funding acquisition, Conceptualization.

## Declaration of competing interest

The authors declare there are no conflicts of interest.

## Data Availability

All data relevant to the study are provided in the article or available as online supplemental material. The data collection form template, along with additional information, can be found in the public research protocol at doi: 10.17605/OSF.IO/TMS26 and in the online supplemental material.

## Appendix A Supplementary data

PRIOR Checklist. Image 1

Study protocol and data extraction form. Image 2

Search strategy, review characteristics, and quality assessment. Image 3

## References

[1] Ramirez V., Montaño F.A., Bautista R.J., Mummidi S., Alvarenga J.C., Bautista C.J. (2021). Lactation plays a fundamental role in developmental programming. Endocr Metab Immune Disord Drug Targets.

[2] Del Ciampo L.A., Del Ciampo I.R.L. (2018). Breastfeeding and the benefits of lactation for Women’s health. Rev Bras Ginecol E Obstet Rev Fed Bras Soc Ginecol E Obstet.

[3] North K., Gao M., Allen G., Lee A.C. (2022). Breastfeeding in a global context: epidemiology, impact, and future directions. Clin Ther.

[4] Victora C.G., Bahl R., Barros A.J.D., França G.V.A., Horton S., Krasevec J. (2016). Lancet breastfeeding series group, breastfeeding in the 21st century: epidemiology, mechanisms, and lifelong effect. Lancet Lond Engl.

[5] Schwarz E.B., Nothnagle M. (2015). The maternal health benefits of breastfeeding. Am Fam Physician.

[6] Veeranki S.P., Nishimura H., Krupp K., Gowda S., Arun A., Madhivanan P. (2017). Suboptimal breastfeeding practices among women in rural and low-resource settings: a study of women in rural Mysore, India. Ann Glob Health.

[7] WHO/UNICEF (2014).

[8] Turke K.C., Santos L.R.D., Matsumura L.S., Sarni R.O.S. (2021). Risk factors for the lack of adherence to breastfeeding. Rev Assoc Med Bras.

[9] Vieira S., Caldeira N.T., Eugênio D.S., di Lucca M.M., Silva I.A. (2018). Breastfeeding self-efficacy and postpartum depression: a cohort study. Rev Lat Am Enfermagem.

[10] Rollins N.C., Bhandari N., Hajeebhoy N., Horton S., Lutter C.K., Martines J.C. (2016). Lancet breastfeeding series group, why invest, and what it will take to improve breastfeeding practices?. Lancet Lond Engl.

[11] Walters D., Eberwein J.D., Sullivan L.M., D’Alimonte M.R., Shekar M. (2016). Reaching the global target to increase exclusive breastfeeding: how much will it cost and how can we pay for it?. Breastfeed Med Off J Acad Breastfeed Med.

[12] Davidove M.E., Dorsey J.W. (2019). Breastfeeding: A cornerstone of healthy sustainable diets. Sustainability.

[13] Salmon L. (2015). Food security for infants and young children: an opportunity for breastfeeding policy?. Int Breastfeed J.

[14] De Souza C.B., Venancio S.I., da Silva R.P.G.V.C. (2021). Breastfeeding support rooms and their contribution to sustainable development goals: A qualitative study. Front Public Health.

[15] Lalama R.A., Bravo Lalama A. (2019). América Latina y los objetivos de desarrollo sostenible: análisis de su viabilidad. Rev Cienc Soc.

[16] Schäpke N., Omann I., Wittmayer J.M., Van Steenbergen F., Mock M. (2017). Linking transitions to sustainability: A study of the societal effects of transition management. Sustainability.

[17] Joffe N., Webster F., Shenker N. (2019). Support for breastfeeding is an environmental imperative. BMJ.

[18] Ortiz M.P., Ortiz E.P. (2007). Health psychology: a key to understand therapeutic adherence. Rev Med Chil.

[19] Dilla T., Valladares A., Lizán L., Sacristán J.A. (2009). Treatment adherence and persistence: causes, consequences and improvement strategies. Aten Primaria.

[20] Huls S.P.I., Whichello C.L., van Exel J., Uyl-de Groot C.A., de Bekker-Grob E.W. (2019). What is next for patient preferences in health technology assessment? A systematic review of the challenges. Value Health J Int Soc Pharmacoeconomics Outcomes Res.

[21] Carrizo E., Domini J., Quezada R.Y.J., Serra S.V., Soria E.A., Miranda A.R. (2020). Variations of the cognitive status in the puerperium and their determinants: a narrative review. Cien Saude Colet.

[22] Gates M., Gates A., Pieper D., Fernandes R.M., Tricco A.C., Moher D. (2022). Reporting guideline for overviews of reviews of healthcare interventions: development of the PRIOR statement. BMJ.

[23] Aromataris E., Fernandez R., Godfrey C.M., Holly C., Khalil H., Tungpunkom P. (2015). Summarizing systematic reviews: methodological development, conduct and reporting of an umbrella review approach. JBI Evid Implement.

[24] Sánchez Martín M., Pedreño Plana M., Ponce Gea A.I., Navarro Mateu F. (2023). Principio, fue la pregunta de investigación …: Los formatos PICO, PECO, SPIDER y FINER. Espiral Cuad. Profr..

[25] Ouzzani M., Hammady H., Fedorowicz Z., Elmagarmid A. (2016). Rayyan-a web and mobile app for systematic reviews. Syst Rev.

[26] Dmytriw A.A., Hui N., Singh T., Nguyen D., Omid-Fard N., Phan K. (2021). Bibliometric evaluation of systematic review and meta analyses published in the top 5 “high-impact” radiology journals. Clin Imaging.

[27] Whiting P., Savović J., Higgins J.P.T., Caldwell D.M., Reeves B.C., Shea B. (2016). ROBIS group, ROBIS: A new tool to assess risk of bias in systematic reviews was developed. J Clin Epidemiol.

[28] Landis J.R., Koch G.G. (1977). The measurement of observer agreement for categorical data. Biometrics.

[29] Thomas J., Harden A. (2008). Methods for the thematic synthesis of qualitative research in systematic reviews. BMC Med Res Methodol.

[30] van Eck N.J., Waltman L. (2010). Software survey: VOSviewer, a computer program for bibliometric mapping. Scientometrics.

[31] Dennis C.-L., Jackson K., Watson J. (2014). Interventions for treating painful nipples among breastfeeding women. Cochrane Database Syst Rev.

[32] Morland-Schultz K., Hill P.D. (2005). Prevention of and therapies for nipple pain: a systematic review. J Obstet Gynecol Neonatal Nurs JOGNN.

[33] Wang Z., Liu Q., Min L., Mao X. (2021). The effectiveness of the laid-back position on lactation-related nipple problems and comfort: a meta-analysis. BMC Pregnancy Childbirth.

[34] O’Shea J.E., Foster J.P., O’Donnell C.P., Breathnach D., Jacobs S.E., Todd D.A. (2017). Frenotomy for tongue-tie in newborn infants. Cochrane Database Syst Rev.

[35] Osadchy A., Moretti M.E., Koren G. (2012). Effect of domperidone on insufficient lactation in puerperal women: a systematic review and meta-analysis of randomized controlled trials. Obstet Gynecol Int.

[36] Grzeskowiak L.E., Smithers L.G., Amir L.H., Grivell R.M. (2018). Domperidone for increasing breast milk volume in mothers expressing breast milk for their preterm infants: a systematic review and meta-analysis. BJOG.

[37] Foong S.C., Tan M.L., Foong W.C., Marasco L.A., Ho J.J., Ong J.H. (2020). Oral galactagogues (natural therapies or drugs) for increasing breast milk production in mothers of non-hospitalised term infants. Cochrane Database Syst Rev.

[38] Dodd J.M., Dare M.R., Middleton P. (2004). Treatment for women with postpartum iron deficiency anaemia. Cochrane Database Syst Rev.

[39] Soubeiga D., Gauvin L., Hatem M.A., Johri M. (2014). Birth preparedness and complication readiness (BPCR) interventions to reduce maternal and neonatal mortality in developing countries: systematic review and meta-analysis. BMC Pregnancy Childbirth.

[40] Fry T.J., Marfurt S., Wengier S. (2018). Systematic review of quality improvement initiatives related to Cue-based feeding in preterm infants. Nurs Womens Health.

[41] Qian J., Wu T., Lv M., Fang Z., Chen M., Zeng Z. (2021). The value of Mobile health in improving breastfeeding outcomes among perinatal or postpartum women: systematic review and Meta-analysis of randomized controlled trials. JMIR Mhealth Uhealth.

[42] Beake S., Bick D., Narracott C., Chang Y.-S. (2017). Interventions for women who have a caesarean birth to increase uptake and duration of breastfeeding: A systematic review. Matern Child Nutr.

[43] Carfoot S., Williamson P.R., Dickson R. (2003). A systematic review of randomised controlled trials evaluating the effect of mother/baby skin-to-skin care on successful breast feeding. Midwifery.

[44] Moore E.R., Bergman N., Anderson G.C., Medley N. (2016). Early skin-to-skin contact for mothers and their healthy newborn infants. Cochrane Database Syst Rev.

[45] Habtewold T.D., Mohammed S.H., Endalamaw A., Akibu M., Sharew N.T., Alemu Y.M. (2019). Breast and complementary feeding in Ethiopia: new national evidence from systematic review and meta-analyses of studies in the past 10 years. Eur J Nutr.

[46] Heesen P., Halpern S.H., Beilin Y., Mauri P.A., Eidelman L.A., Heesen M. (2021). Labor neuraxial analgesia and breastfeeding: an updated systematic review. J Clin Anesth.

[47] Getaneh T., Negesse A., Dessie G., Desta M., Temesgen H., Getu T. (2021). Impact of cesarean section on timely initiation of breastfeeding in Ethiopia: a systematic review and meta-analysis. Int Breastfeed J.

[48] Prior E., Santhakumaran S., Gale C., Philipps L.H., Modi N., Hyde M.J. (2012). Breastfeeding after cesarean delivery: a systematic review and meta-analysis of world literature. Am J Clin Nutr.

[49] Patil D.S., Pundir P., Dhyani V.S., Krishnan J.B., Parsekar S.S., D’Souza S.M. (2020). A mixed-methods systematic review on barriers to exclusive breastfeeding. Nutr Health.

[50] Chang Y.-S., Glaria A.A., Davie P., Beake S., Bick D. (2020). Breastfeeding experiences and support for women who are overweight or obese: A mixed-methods systematic review. Matern Child Nutr.

[51] Miao Y., Zhao S., Liu W., Jiang H., Li Y., Wang A. (2023). Prevalence and risk factors of delayed onset lactogenesis II in China: a systematic review and meta-analysis. J Matern-Fetal Neonatal Med Off J Eur Assoc Perinat Med Fed Asia Ocean Perinat Soc Int Soc Perinat Obstet.

[52] Douglas P.S., Hill P.S. (2013). Behavioral sleep interventions in the first six months of life do not improve outcomes for mothers or infants: a systematic review. J Dev Behav Pediatr JDBP.

[53] Allen E., Rumbold A.R., Keir A., Collins C.T., Gillis J., Suganuma H. (2021). Avoidance of bottles during the establishment of breastfeeds in preterm infants. Cochrane Database Syst Rev.

[54] Flint A., New K., Davies M.W. (2016). Cup feeding versus other forms of supplemental enteral feeding for newborn infants unable to fully breastfeed. Cochrane Database Syst Rev.

[55] Davie P., Chilcot J., Chang Y.-S., Norton S., Hughes L.D., Bick D. (2020). Effectiveness of social-psychological interventions at promoting breastfeeding initiation, duration and exclusivity: a systematic review and meta-analysis, health. Psychol Rev.

[56] Graves L.E., Turner S., Nader M., Sinha S. (2016). Breastfeeding and opiate substitution therapy: starting to understand infant feeding choices. Subst Abuse Res Treat.

[57] Ndikom C.M., Fawole B., Ilesanmi R.E. (2014). Extra fluids for breastfeeding mothers for increasing milk production. Cochrane Database Syst Rev.

[58] Gallos I.D., Papadopoulou A., Man R., Athanasopoulos N., Tobias A., Price M.J. (2018). Uterotonic agents for preventing postpartum haemorrhage: a network meta-analysis. Cochrane Database Syst Rev.

[59] Kapp N., Curtis K.M. (2010). Combined oral contraceptive use among breastfeeding women: a systematic review. Contraception.

[60] Lopez L.M., Grey T.W., Stuebe A.M., Chen M., Truitt S.T., Gallo M.F. (2015). Combined hormonal versus nonhormonal versus progestin-only contraception in lactation. Cochrane Database Syst Rev.

[61] Carr S.L., Gaffield M.E., Dragoman M.V., Phillips S. (2016). Safety of the progesterone-releasing vaginal ring (PVR) among lactating women: A systematic review. Contraception.

[62] Sothornwit J., Kaewrudee S., Lumbiganon P., Pattanittum P., Averbach S.H. (2022). Immediate versus delayed postpartum insertion of contraceptive implant and IUD for contraception. Cochrane Database Syst Rev.

[63] Carter E.B., Temming L.A., Akin J., Fowler S., Macones G.A., Colditz G.A. (2016). Group prenatal care compared with traditional prenatal care: A systematic review and Meta-analysis. Obstet Gynecol.

[64] Jaafar S.H., Ho J.J., Lee K.S. (2016). Rooming-in for new mother and infant versus separate care for increasing the duration of breastfeeding. Cochrane Database Syst Rev.

[65] Jones E., Stewart F., Taylor B., Davis P.G., Brown S.J. (2021). Early postnatal discharge from hospital for healthy mothers and term infants. Cochrane Database Syst Rev.

[66] Thibaudeau S., Sinno H., Williams B. (2010). The effects of breast reduction on successful breastfeeding: a systematic review. J Plast Reconstr Aesthetic Surg JPRAS.

[67] Nguyen G.C., Seow C.H., Maxwell C., Huang V., Leung Y., Jones J. (2016). IBD in pregnancy consensus group, Canadian Association of Gastroenterology, the Toronto consensus statements for the Management of Inflammatory Bowel Disease in pregnancy. Gastroenterology.

[68] Oliveira M.R.E., Santos M.G., Aude D.A., Lima R.M.E., Módolo N.S.P., Navarro L.H. (2019). Should maternal anesthesia delay breastfeeding? A systematic review of the literature. Braz J Anesthesiol Elsevier.

[69] French C.A., Cong X., Chung K.S. (2016). Labor epidural analgesia and breastfeeding: A systematic review. J Hum Lact.

[70] Truitt S.T., Fraser A.B., Grimes D.A., Gallo M.F., Schulz K.F. (2003). Hormonal contraception during lactation. Systematic review of randomized controlled trials. Contraception.

[71] McDonagh M.S., Matthews A., Phillipi C., Romm J., Peterson K., Thakurta S. (2014). Depression drug treatment outcomes in pregnancy and the postpartum period: a systematic review and meta-analysis. Obstet Gynecol.

[72] Brixval C.S., Axelsen S.F., Lauemøller S.G., Andersen S.K., Due P., Koushede V. (2015). The effect of antenatal education in small classes on obstetric and psycho-social outcomes - a systematic review. Syst Rev.

[73] Johns H.M., Forster D.A., Amir L.H., McLachlan H.L. (2013). Prevalence and outcomes of breast milk expressing in women with healthy term infants: a systematic review. BMC Pregnancy Childbirth.

[74] Wouk K., Tully K.P., Labbok M.H. (2017). Systematic review of evidence for baby-friendly hospital initiative step 3. J Hum Lact Off J Int Lact Consult Assoc.

[75] Skouteris H., Bailey C., Nagle C., Hauck Y., Bruce L., Morris H. (2017). Interventions designed to promote exclusive breastfeeding in high-income countries: A systematic review update. Breastfeed Med Off J Acad Breastfeed Med.

[76] Hall Moran V., Edwards J., Dykes F., Downe S. (2007). A systematic review of the nature of support for breast-feeding adolescent mothers. Midwifery.

[77] Wu W., Zhang J., Silva Zolezzi I., Fries L.R., Zhao A. (2021). Factors influencing breastfeeding practices in China: A meta-aggregation of qualitative studies, Matern. Child Nutr.

[78] Nelson A.M. (2006). A metasynthesis of qualitative breastfeeding studies. J Midwifery Womens Health.

[79] McFadden A., Siebelt L., Marshall J.L., Gavine A., Girard L.-C., Symon A. (2019). Counselling interventions to enable women to initiate and continue breastfeeding: a systematic review and meta-analysis. Int Breastfeed J.

[80] Beake S., Pellowe C., Dykes F., Schmied V., Bick D. (2011). A systematic review of structured versus non-structured breastfeeding programmes to support the initiation and duration of exclusive breastfeeding in acute and primary healthcare settings. JBI Libr Syst Rev.

[81] Fallon V.M., Harrold J.A., Chisholm A. (2019). The impact of the UK baby friendly initiative on maternal and infant health outcomes: A mixed-methods systematic review. Matern Child Nutr.

[82] Schmied V., Thomson G., Byrom A., Burns E., Sheehan A., Dykes F. (2014). A meta-ethnographic study of health care staff perceptions of the WHO/UNICEF baby friendly health initiative, women birth J. Aust. Coll. Midwives.

[83] Balogun O.O., O’Sullivan E.J., McFadden A., Ota E., Gavine A., Garner C.D. (2016). Interventions for promoting the initiation of breastfeeding. Cochrane Database Syst Rev.

[84] Patel S., Patel S. (2016). The effectiveness of lactation consultants and lactation counselors on breastfeeding outcomes. J Hum Lact Off J Int Lact Consult Assoc.

[85] Wood N.K., Woods N.F., Blackburn S.T., Sanders E.A. (2016). Interventions that enhance breastfeeding initiation, duration, and exclusivity: A systematic review. MCN: Am J Matern Child Nurs.

[86] Russell P.S., Smith D.M., Birtel M.D., Hart K.H., Golding S.E. (2022). The role of emotions and injunctive norms in breastfeeding: a systematic review and meta-analysis, health. Psychol Rev.

[87] Doerzbacher M., Chang Y.-P. (2019). Supporting breastfeeding for women on opioid maintenance therapy: a systematic review. J Perinatol Off J Calif Perinat Assoc.

[88] Mangham-Jefferies L., Pitt C., Cousens S., Mills A., Schellenberg J. (2014). Cost-effectiveness of strategies to improve the utilization and provision of maternal and newborn health care in low-income and lower-middle-income countries: a systematic review. BMC Pregnancy Childbirth.

[89] Brockway M., Benzies K., Hayden K.A. (2017). Interventions to improve breastfeeding self-efficacy and resultant breastfeeding rates: A systematic review and Meta-analysis. J Hum Lact Off J Int Lact Consult Assoc.

[90] Olufunlayo T.F., Roberts A.A., MacArthur C., Thomas N., Odeyemi K.A., Price M. (2019). Improving exclusive breastfeeding in low and middle-income countries: A systematic review. Matern Child Nutr.

[91] Yonemoto N., Nagai S., Mori R. (2021). Schedules for home visits in the early postpartum period. Cochrane Database Syst Rev.

[92] Scott K.D., Klaus P.H., Klaus M.H. (1999). The obstetrical and postpartum benefits of continuous support during childbirth. J Womens Health Gend Based Med.

[93] Bohren M.A., Hofmeyr G.J., Sakala C., Fukuzawa R.K., Cuthbert A. (2017). Continuous support for women during childbirth. Cochrane Database Syst Rev.

[94] Sandall J., Turienzo C.F., Devane D., Soltani H., Gillespie P., Gates S. (2024). Midwife continuity of care models versus other models of care for childbearing women. Cochrane Database Syst Rev.

[95] Meedya S., Fahy K., Kable A. (2010). Factors that positively influence breastfeeding duration to 6 months: a literature review, women birth J. Aust. Coll. Midwives.

[96] Seward N., Neuman M., Colbourn T., Osrin D., Lewycka S., Azad K. (2017). Effects of women’s groups practising participatory learning and action on preventive and care-seeking behaviours to reduce neonatal mortality: A meta-analysis of cluster-randomised trials. PLoS Med.

[97] Morse H., Brown A. (2022). The benefits, challenges and impacts of accessing social media group support for breastfeeding: A systematic review. Matern Child Nutr.

[98] Fairbank L., O’Meara S., Renfrew M.J., Woolridge M., Sowden A.J., Lister-Sharp D. (2000). A systematic review to evaluate the effectiveness of interventions to promote the initiation of breastfeeding. Health Technol Assess (Winch Eng).

[99] Hunt L., Thomson G., Whittaker K., Dykes F. (2022). Non-profit breastfeeding organisations’ peer support provision in areas of socio-economic deprivation in the UK: A meta-ethnography. Matern Child Nutr.

[100] Gavine A., Shinwell S.C., Buchanan P., Farre A., Wade A., Lynn F. (2022). Support for healthy breastfeeding mothers with healthy term babies. Cochrane Database Syst Rev.

[101] Huang Y., Liu Y., Yu X.-Y., Zeng T.-Y. (2022). The rates and factors of perceived insufficient milk supply: A systematic review. Matern Child Nutr.

[102] Wong M.S., Mou H., Chien W.T. (2021). Effectiveness of educational and supportive intervention for primiparous women on breastfeeding related outcomes and breastfeeding self-efficacy: A systematic review and meta-analysis. Int J Nurs Stud.

[103] Lavender T., Richens Y., Milan S.J., Smyth R.M.D., Dowswell T. (2013). Telephone support for women during pregnancy and the first six weeks postpartum. Cochrane Database Syst Rev.

[104] Arikpo D., Edet E.S., Chibuzor M.T., Odey F., Caldwell D.M. (2018). Educational interventions for improving primary caregiver complementary feeding practices for children aged 24 months and under. Cochrane Database Syst Rev.

[105] Alahmed S., Meedya S., Mutair A.A., Fernandez R. (2023). Saudi Women’s breastfeeding knowledge, attitude, and practices: A systematic review and Meta-analysis. J Transcult Nurs.

[106] Gyamfi A., Spatz D.L., Jefferson U.T., Lucas R., O’Neill B., Henderson W.A. (2023). Breastfeeding social support among African American women in the United States: A Meta-ethnography. Adv Neonatal Care Off J Natl Assoc Neonatal Nurses.

[107] Higginbottom G.M.A., Morgan M., Alexandre M., Chiu Y., Forgeron J., Kocay D. (2015). Immigrant women’s experiences of maternity-care services in Canada: a systematic review using a narrative synthesis. Syst Rev.

[108] Baddock S.A., Purnell M.T., Blair P.S., Pease A.S., Elder D.E., Galland B.C. (2019). The influence of bed-sharing on infant physiology, breastfeeding and behaviour: A systematic review. Sleep Med Rev.

[109] Lumbiganon P., Martis R., Laopaiboon M., Festin M.R., Ho J.J., Hakimi M. (2016). Antenatal breastfeeding education for increasing breastfeeding duration. Cochrane Database Syst Rev.

[110] Vitalis D., Vilar-Compte M., Nyhan K., Pérez-Escamilla R. (2021). Breastfeeding inequities in South Africa: can enforcement of the WHO code help address them? - A systematic scoping review. Int J Equity Health.

[111] Da Silva Tanganhito D., Bick D., Chang Y.-S. (2020). Breastfeeding experiences and perspectives among women with postnatal depression: A qualitative evidence synthesis, women birth J. Aust. Coll. Midwives.

[112] Whitford H.M., Wallis S.K., Dowswell T., West H.M., Renfrew M.J. (2017). Breastfeeding education and support for women with twins or higher order multiples. Cochrane Database Syst Rev.

[113] Vilar-Compte M., Hernández-Cordero S., Ancira-Moreno M., Burrola-Méndez S., Ferre-Eguiluz I., Omaña I. (2021). Breastfeeding at the workplace: a systematic review of interventions to improve workplace environments to facilitate breastfeeding among working women. Int J Equity Health.

[114] Kim J.H., Shin J.C., Donovan S.M. (2019). Effectiveness of workplace lactation interventions on breastfeeding outcomes in the United States: an updated systematic review. J Hum Lact Off J Int Lact Consult Assoc.

[115] Dinour L.M., Szaro J.M. (2017). Employer-based programs to support breastfeeding among working mothers: A systematic review. Breastfeed Med Off J Acad Breastfeed Med.

[116] Hirani S.A.A., Karmaliani R. (2013). Evidence based workplace interventions to promote breastfeeding practices among Pakistani working mothers, women birth J. Aust. Coll. Midwives.

[117] Owens B.A., DiTomasso D. (2024). Practices and policies that support breastfeeding among military women: A systematic review. Mil Med.

[118] Turcksin R., Bel S., Galjaard S., Devlieger R. (2014). Maternal obesity and breastfeeding intention, initiation, intensity and duration: a systematic review. Matern Child Nutr.

[119] Garcia A.H., Voortman T., Baena C.P., Chowdhurry R., Muka T., Jaspers L. (2016). Maternal weight status, diet, and supplement use as determinants of breastfeeding and complementary feeding: a systematic review and meta-analysis. Nutr Rev.

[120] Amir L.H., Donath S. (2007). A systematic review of maternal obesity and breastfeeding intention, initiation and duration. BMC Pregnancy Childbirth.

[121] Fair F.J., Ford G.L., Soltani H. (2019). Interventions for supporting the initiation and continuation of breastfeeding among women who are overweight or obese. Cochrane Database Syst Rev.

[122] Reichental Z.L., O’Brien V.M., O’Reilly S.L. (2022). Interventions to support women with overweight or obesity or gestational diabetes mellitus to initiate and continue breastfeeding: systematic review and meta-analysis. Obes Rev Off J Int Assoc Study Obes.

[123] Taylor J.S., Kacmar J.E., Nothnagle M., Lawrence R.A. (2005). A systematic review of the literature associating breastfeeding with type 2 diabetes and gestational diabetes. J Am Coll Nutr.

[124] Manerkar K., Harding J., Conlon C., McKinlay C. (2020). Maternal gestational diabetes and infant feeding, nutrition and growth: a systematic review and meta-analysis. Br J Nutr.

[125] Lyons S., Currie S., Peters S., Lavender T., Smith D.M. (2018). The association between psychological factors and breastfeeding behaviour in women with a body mass index (BMI) ≥30 kg m-2 : a systematic review. Obes Rev Off J Int Assoc Study Obes.

[126] Badr H.A., Zauszniewski J.A. (2017). Meta-analysis of the predictive factors of postpartum fatigue. Appl Nurs Res ANR.

[127] Butler M.S., Young S.L., Tuthill E.L. (2021). Perinatal depressive symptoms and breastfeeding behaviors: A systematic literature review and biosocial research agenda. J Affect Disord.

[128] Dennis C.-L., McQueen K. (2009). The relationship between infant-feeding outcomes and postpartum depression: a qualitative systematic review. Pediatrics.

[129] Oyetunji A., Chandra P. (2020). Postpartum stress and infant outcome: A review of current literature. Psychiatry Res.

[130] Baker N., Bick D., Bamber L., Wilson C.A., Howard L.M., Bakolis I. (2023). A mixed methods systematic review exploring infant feeding experiences and support in women with severe mental illness. Matern Child Nutr.

[131] de Jager E., Skouteris H., Broadbent J., Amir L., Mellor K. (2013). Psychosocial correlates of exclusive breastfeeding: a systematic review. Midwifery.

[132] Kimmel M.C., Ferguson E.H., Zerwas S., Bulik C.M., Meltzer-Brody S. (2016). Obstetric and gynecologic problems associated with eating disorders. Int J Eat Disord.

[133] Kaß A., Dörsam A.F., Weiß M., Zipfel S., Giel K.E. (2021). The impact of maternal eating disorders on breastfeeding practices: a systematic review. Arch Womens Ment Health.

[134] Grant A., Jones S., Williams K., Leigh J., Brown A. (2022). Autistic women’s views and experiences of infant feeding: A systematic review of qualitative evidence. Autism Int J Res Pract.

[135] John G.C., Richardson B.A., Nduati R.W., Mbori-Ngacha D., Kreiss J.K. (2001). Timing of breast milk HIV-1 transmission: a meta-analysis, east Afr. Medizinhist J.

[136] Antoniou E., Andronikidi P.E., Eskitzis P., Iliadou M., Palaska E., Tzitiridou-Chatzopoulou M. (2023). Congenital Zika syndrome and disabilities of feeding and breastfeeding in early childhood: A systematic review. Viruses.

[137] Bhurosy T., Niu Z., Heckman C.J. (2021). Breastfeeding is possible: A systematic review on the feasibility and challenges of breastfeeding among breast Cancer survivors of reproductive age. Ann Surg Oncol.

[138] Gray R., Bick D., Chang Y.-S. (2014). Health in pregnancy and post-birth: contribution to improved child outcomes. J Child Serv.

[139] Conde-Agudelo A., Rosas-Bermudez A., Castaño F., Norton M.H. (2012). Effects of birth spacing on maternal, perinatal, infant, and child health: a systematic review of causal mechanisms. Stud Fam Plann.

[140] Bruney T.L., Scime N.V., Madubueze A., Chaput K.H. (2022). Systematic review of the evidence for resolution of common breastfeeding problems-Ankyloglossia (tongue tie). Acta Paediatr Oslo Nor.

[141] Dalili H., Shariat M., Nayeri F., Emami Z., Sahebi R., Sahebi L. (2020). Duration of breastfeeding and maternal-related factors in Iran, systematic review and Meta-analysis. J Pediatr Nurs.

[142] Adams I.K.R., Okoli C.T.C., Dulin Keita A., Linares A.M., Tanaka K., Polanin J.R. (2016). Breastfeeding practices among native Hawaiians and Pacific islanders. J Obes.

[143] Hedberg I.C. (2013). Barriers to breastfeeding in the WIC population. MCN: Am J Matern Child Nurs.

[144] Robinson K., Fial A., Hanson L. (2019). Racism, Bias, and discrimination as modifiable barriers to breastfeeding for African American women: A scoping review of the literature. J Midwifery Womens Health.

[145] Jones K.M., Power M.L., Queenan J.T., Schulkin J. (2015). Racial and ethnic disparities in breastfeeding. Breastfeed Med Off J Acad Breastfeed Med.

[146] Johnson A., Kirk R., Rosenblum K.L., Muzik M. (2015). Enhancing breastfeeding rates among African American women: a systematic review of current psychosocial interventions. Breastfeed Med Off J Acad Breastfeed Med.

[147] Springall T.L., McLachlan H.L., Forster D.A., Browne J., Chamberlain C. (2023). Factors associated with breastfeeding initiation and maintenance for aboriginal and Torres Strait islander women in Australia: A systematic review and narrative analysis, women birth J. Aust. Coll. Midwives.

[148] Springall T.L., McLachlan H.L., Forster D.A., Browne J., Chamberlain C. (2022). Breastfeeding rates of aboriginal and Torres Strait islander women in Australia: a systematic review and narrative analysis, women birth J. Aust. Coll. Midwives.

[149] Mitchell F., Walker T., Hill K., Browne J. (2023). Factors influencing infant feeding for aboriginal and Torres Strait islander women and their families: a systematic review of qualitative evidence. BMC Public Health.

[150] Dennis C.L., Shiri R., Brown H.K., Santos H.P., Schmied V., Falah-Hassani K. (2019). Breastfeeding rates in immigrant and non-immigrant women: A systematic review and meta-analysis. Matern Child Nutr.

[151] Ratnayake Mudiyanselage S., Davis D., Kurz E., Atchan M. (2022). Infant and young child feeding during natural disasters: A systematic integrative literature review, women birth J. Aust. Coll. Midwives.

[152] Adesanya A.M., Barrett S., Moffat M., Aquino M.R.J., Nicholson W., Turner G. (2022). Impact of the COVID-19 pandemic on expectant and new parents’ experience of pregnancy, childbirth, breast feeding, parental responsiveness and sensitivity, and bonding and attunement in high-income countries: a systematic review of the evidence. BMJ Open.

[153] Segura-Pérez S., Hromi-Fiedler A., Adnew M., Nyhan K., Pérez-Escamilla R. (2021). Impact of breastfeeding interventions among United States minority women on breastfeeding outcomes: a systematic review. Int J Equity Health.

[154] Andersen M.Z., Zeinert P., Rosenberg J., Fonnes S. (2024). Comparative analysis of Cochrane and non-Cochrane reviews over three decades. Syst Rev.

[155] Gebresilassie K.Y., Baraki A.G., Kassie B.A., Wami S.D. (2022). Midwifery-led researches for evidence-based practice: clinical midwives engagement in research in Ethiopia, 2021. PloS One.

[156] Bonilla H., Ortiz-Llorens M., Barger M.K., Rodríguez C., Cabrera M. (2018). Implementation of a programme to develop research projects in a school of midwifery in Santiago, Chile. Midwifery.

[157] Nicholls L., Webb C. (2025). What makes a good midwife? An integrative review of methodologically-diverse research. http://dx.doi.org/10.1111/j.1365-2648.2006.04026.x.

[158] McNeill J., Lynn F., Alderdice F. (2012). Public health interventions in midwifery: a systematic review of systematic reviews. BMC Public Health.

[159] De Leo A., Bayes S., Geraghty S., Butt J. (2019). Midwives’ use of best available evidence in practice: an integrative review. J Clin Nurs.

[160] Sabancı Baransel E., Uçar T., Çelik O.T. (2023). Mapping publication status and exploring hotspots in a research field: breastfeeding. J Hum Lact Off J Int Lact Consult Assoc.

[161] Marom R., Lubetzky R., Mimouni F.B., Ovental A., Mandel D., Cohen S. (2014). Secular trends in impact factor of breastfeeding research publications over a 20-year period. Breastfeed Med Off J Acad Breastfeed Med.

[162] Asiri F.Y., Kruger E., Tennant M. (2021). The top 100 Most cited articles published in dentistry: 2020 update. Healthc Basel Switz.

[163] Belter C.W. (2015). Bibliometric indicators: opportunities and limits. J Med Libr Assoc JMLA.

[164] Aksnes D.W., Langfeldt L., Wouters P. (2019). Citations, citation indicators, and research quality: an overview of basic concepts and theories. Sage Open.

[165] Luong V., Bearman M., MacLeod A. (2023). Understanding Meta-ethnography in health professions education research. J Grad Med Educ.

[166] Gough D. (2015). Qualitative and mixed methods in systematic reviews. Syst Rev.

[167] Sattar R., Lawton R., Panagioti M., Johnson J. (2021). Meta-ethnography in healthcare research: a guide to using a meta-ethnographic approach for literature synthesis. BMC Health Serv Res.

[168] Noyes J., Booth A., Moore G., Flemming K., Tunçalp Ö., Shakibazadeh E. (2019). Synthesising quantitative and qualitative evidence to inform guidelines on complex interventions: clarifying the purposes, designs and outlining some methods. BMJ Glob Health.

[169] Stern C., Lizarondo L., Carrier J., Godfrey C., Rieger K., Salmond S. (2020). Methodological guidance for the conduct of mixed methods systematic reviews. JBI Evid Synth.

[170] Hawkins M.M., Schmitt M.E., Adebayo C.T., Weitzel J., Olukotun O., Christensen A.M. (2021). Promoting the health of refugee women: a scoping literature review incorporating the social ecological model. Int J Equity Health.

[171] McGinnis M.D., Ostrom E. (2014). Social-ecological system framework: initial changes and continuing challenges. Ecol Soc.

[172] Levin S., Xepapadeas T., Crépin A.-S., Norberg J., de Zeeuw A., Folke C. (2013). Social-ecological systems as complex adaptive systems: modeling and policy implications. Environ Dev Econ.

[173] Abekah-Nkrumah G., Antwi M.Y., Nkrumah J., Gbagbo F.Y. (2020). Examining working mothers’ experience of exclusive breastfeeding in Ghana. Int Breastfeed J.

[174] Snyder K., Hulse E., Dingman H., Cantrell A., Hanson C., Dinkel D. (2021). Examining supports and barriers to breastfeeding through a socio-ecological lens: a qualitative study. Int Breastfeed J.

[175] Ingram L., MacArthur C., Khan K., Deeks J.J., Jolly K. (2010). Effect of antenatal peer support on breastfeeding initiation: a systematic review. CMAJ.

[176] Kummer L., Duke N., Davis L., Borowsky I. (2020). Association of Social and Community Factors with U.S. breastfeeding outcomes. Breastfeed Med Off J Acad Breastfeed Med.

[177] Jolly K., Ingram L., Khan K.S., Deeks J.J., Freemantle N., MacArthur C. (2012). Systematic review of peer support for breastfeeding continuation: metaregression analysis of the effect of setting, intensity, and timing. BMJ.

[178] Nsiah-Asamoah C., Doku D.T., Agblorti S. (2020). Mothers’ and Grandmothers’ misconceptions and socio-cultural factors as barriers to exclusive breastfeeding: A qualitative study involving health workers in two rural districts of Ghana. PloS One.

[179] Shittu F., King C., Rautiainen S., Iuliano A., Bakare A.A., Colbourn T. (2024). Exploring the feeding practices of mothers of under-five children and how household members influence exclusive breastfeeding in Jigawa state, Nigeria – A qualitative study. Glob Public Health.

[180] Wanjohi M., Griffiths P., Wekesah F., Muriuki P., Muhia N., Musoke R.N. (2017). Sociocultural factors influencing breastfeeding practices in two slums in Nairobi, Kenya. Int Breastfeed J.

[181] Paramashanti B.A., Dibley M.J., Huda T.M., Alam A. (2022). Breastfeeding perceptions and exclusive breastfeeding practices: A qualitative comparative study in rural and urban Central Java, Indonesia. Appetite.

[182] Llorente-Pulido S., Custodio E., López-Giménez M.R., Sanz-Barbero B., Otero-García L. (2021). Barriers and facilitators for exclusive breastfeeding in Women’s biopsychosocial spheres according to primary care midwives in Tenerife (Canary Islands, Spain). Int J Environ Res Public Health.

[183] Green V.L., Killings N.L., Clare C.A. (2021). The historical, psychosocial, and cultural context of breastfeeding in the African American community. Breastfeed Med.

[184] Phonyiam R., Berry D.C. (2021). Facilitators and barriers to breastfeeding in Asian American women: A review of the literature. Asian J Pregnancy Childbirth.

[185] Monteith H., Checholik C., Galloway T., Sahak H., Shawanda A., Liu C. (2024). Infant feeding experiences among indigenous communities in Canada, the United States, Australia, and Aotearoa: a scoping review of the qualitative literature. BMC Public Health.

[186] Choi G.J., Kang H. (2023). Introduction to umbrella reviews as a useful evidence-based practice. J Lipid Atheroscler.

[187] Goossen K., Hess S., Lunny C., Pieper D. (2020). Database combinations to retrieve systematic reviews in overviews of reviews: a methodological study. BMC Med Res Methodol.

[188] Kumar R.P., Perumpully S.J., Samuel C., Gautam S. (2023). Exposure and health: A progress update by evaluation and scientometric analysis. Stoch Environ Res Risk Assess Res J.

[189] Paez A. (2017). Gray literature: an important resource in systematic reviews. J Evid-Based Med.

[190] Mansournia G.S. Collins, Nielsen R.O., Nazemipour M., Jewell N.P., Altman D.G., Campbell M.J. (2021). A CHecklist for statistical assessment of medical papers (the CHAMP statement): explanation and elaboration. Br J Sports Med.

[191] Archibald M., Wiebe S., Rieger K., Linton J., Woodgate R. (2021). Protocol for a systematic review of living labs in healthcare. BMJ Open.

