# Supplementary material for: An overview of reviews of breastfeeding barriers and facilitators: Analyzing global research trends and hotspots
Source: Glob Epidemiol. 2025 Mar 6;9:100192. doi: 10.1016/j.gloepi.2025.100192 (PMC11931314; doi:10.1016/j.gloepi.2025.100192)
Supplement: Supplementary file 2 — Study protocol and data extraction form. [file mmc2.pdf]

Protocol: Facilitators and barriers influencing breastfeeding outcomes: an umbrella review.

Contributors:

1. Agustín Ramiro Miranda
2. Paula Eugenia Barral
3. Mariela Valentina Cortez
4. Ana Veronica Scotta
5. Elio Andrés Soria

Date created: 2022-10-06

Identifier: DOI 10.17605/OSF.IO/TMS26

Category: Project

Description: *This umbrella review (UR) will be conducted based on the methodology of UR of multiple systematic reviews. It was undertaken through systematic synthesis of the eligible systematic reviews and meta-analysis (SRM) reports on breastfeeding and its determinants.*

License: *CC-By Attribution 4.0 International*

Facilitators and barriers influencing breastfeeding outcomes: an umbrella review.

Study Information

Hypotheses

This UR focuses on the hypothesis that breastfeeding outcomes are determined by health status, biological, behavioral, enviromental, health care, and social factors.

Design Plan

Study type

Meta-Analysis - A systematic review of published studies.

Blinding

No blinding is involved in this study.

Is there any additional blinding in this study?

Blinding will be used during the article selection and quality assessment processes.

Study design

This UR aims to (a) compile all existing review evidence on determinants of breastfeeding outcomes and to determine how best to translate the evidence; (b) report the effects of genetic, behavioral, environmental, physical, health care, and social factors on breastfeeding outcomes; (c) to address the quality, strengths and limitations of review evidence; and (d) to identify current gaps in the literature and make suggestions for future research.
Adapted PICOS criteria were used to define the characteristics of the studies included in this study:
Participants/population: The population of interest was defined as lactating people with no restrictions based on socio-demographic factors, health status and reproductive characteristics.
Phenomena of interest: Reviews aimed to identify barriers and facilitators of lactation practice amongst lactating people.
Context/setting: Reviews regarding any type of care context/setting including community, primary health care or acute care, any type of living or geographical settings.
Outcomes: Reviews reporting associations of barriers and facilitators with lactation duration, cessation, discontinuation, exclusive lactation, and self-efficacy were included.
Types of studies: systematic review and/or meta-analysis design.

No files selected

Randomization

No response

Sampling Plan

Existing Data

Registration prior to creation of data

Explanation of existing data

No response

Data collection procedures

Seven international online databases (PubMed, Scopus, EBSCO, BVS, data bases specific to systematic reviews such as the Cochrane Database of Systematic Reviews, BMC systematic reviews, and Health evidence) will be searched for SRM studies on breastfeeding determinants. For accessing relevant data about breastfeeding, a comprehensive search will be conducted through the aforementioned databases using adapted PICOS question. This question is developed from the following search key words and/or Medical Subject Headings (MeSH) which were combined using the “OR” and “AND” Boolean operators: ((newborn) OR (neonate) OR (infant)) AND ((postpartum woman) OR (postnatal woman) OR (puerperal woman) OR (lactating woman)) AND ((breastfeeding) OR (lactation) OR (breast feeding) OR (chestfeeding)) AND ((determinants) OR (barriers) OR (facilitators) OR (factors) OR (inequities) OR (disparities) OR (beliefs) OR (myths)) AND ((discontinuation) OR (cessation) OR (interruption) OR (termination) OR (duration) OR (latching) OR (self-efficacy) OR (failure) OR (initiation) OR (supply) OR (sucking) OR (suction) OR (suckling) OR (bonding) OR (attachment) OR (hypogalactia) OR (lactation insufficiency) OR (insufficient milk syndrome) OR (agalactia) OR (agalactorrhea) OR (hypogalactorrhea) OR (breast pain) OR (nipple pain) OR (discomfort)) AND ((Meta-Analysis) OR (Systematic Review))

Literature search will be conducted from October 15/2022 until November 15/2022. The literature search will be performed by three independent researchers, with discrepancies resolved by discussion and consensus.

No files selected

Sample size

Not applicable, this is an umbrella review.

Sample size rationale

No response

Stopping rule

No response

Variables

Manipulated variables

No response

No files selected

Measured variables

- Bibliometric variables
- Bibliographical variables
- Risk of bias (quality) assessment
- Facilitators/enablers and barriers identified by study

No files selected

Indices

No response

No files selected

Analysis Plan

Statistical models

The facilitators and barriers will be synthesized by quantitative and qualitative methods, specifically thematic analysis.

No files selected

Transformations

No response

Inference criteria

No response

Data exclusion

No response

Missing data

No response

Exploratory analysis

No response

Other

Other

No response

Copyright © 2011-2024 Center for Open Science | Terms of Use | Privacy Policy | Status | API
TOP Guidelines | Reproducibility Project: Psychology | Reproducibility Project: Cancer Biology

# Formulario de extracción de datos: Facilitators and barriers influencing breastfeeding outcomes: an umbrella review.

* Indica que la pregunta es obligatoria

1. Nombre (Ana, Agustín o Paula) *

Datos bibliométricos

2. Apellido del primer autor. Cuando sean dos, colocar el de ambos. Si son más, colocar et al. Ej.: *
   - Scotta
   - Scotta and Barral
   - Scotta et al.

3. Año de publicación del artículo *

4. Revista *

5. País de residencia del autor de correspondencia *

6. Cuartil más alto de la revista (para el año de publicación) *

   Marca solo un óvalo.

   - Q1
   - Q2
   - Q3
   - Q4
   - Sin Q

7. IF de la revista (para el año de publicación) *
   Consultar en https://www.scijournal.org/ (columna **2-year**)

8. Open access *

   Marca solo un óvalo.

   - Si
   - No

Datos metodológicos

9. Objetivo/s de la revisión. (Si son más de uno, enlistar con formato "1)"). *

10. Número de estudios incluídos en la revisión (después de la depuración). *

11. Tipo de revisión *

Marca solo un óvalo.

- ( ) Revisión sistemática
- ( ) Meta-análisis
- ( ) Meta-etnografía
- ( ) Mixta
- ( ) Otra (de alcance, síntesis rápida, etc.)

12. Setting (por ejemplo, hospital, centro de atención primaria, rural, país, región, mundial). *

13. Idiomas de los artículos revisados

Selecciona todos los que correspondan.

	Si
**Inglés**	☐
**Español**	☐
**Portugués**	☐
**Francés**	☐
**Chino mandarín**	☐
**Árabe**	☐
**Otro**	☐
**No restringe por idioma**	☐

14. Bases de datos utilizadas para las búsquedas *

15. Rango temporal de búsqueda aplicado. Si no restringen la búsqueda en una ventana tempora, colocar "no restringe por año". *

16. Diseños de los estudios revisados. Colocar una lista con el diseño metodológico y en n de artículos que cumplen con ese diseño. Ej.: *
Transversales (n = 2)
Casos y controles (n = 1)
Ensayos clínicos aleatorizados (n = 10)
Ensayos clínicos no aleatorizados (n = 7)

17. Rango del tamaño de muestra total de la revisión (es decir, la cantidad de personas de los estudios primarios revisados). Ejemplo.: 25 - 1200 *

18. Rango de edad o media/mediana de los estudios primarios. Ejemplo.: 16 - 45. *

19. País de origen de los estudios incluidos. Ej.: USA (n = 3), UK (n = 1), Australia (n = 7) *

20. Instrumentos utilizados para la evaluación de la calidad/riesgo de sesgo. *

Resultados de la revisión

21. Medidas de resultado. Ejemplo: duración de lactancia, inicio de lactancia, abandono de lactancia, etc. *

22. Barreras/factores negativos evaluados. Ejemplo: depresión postparto, acceso, cesárea, etc. *

23. Facilitadores/factores positivos evaluados. Ejemplo: programa sanitario, nivel educativo, información, etc.

24. Resultados generales *

25. Significancia/direccionalidad. *

26. Heterogeneidad (SOLO PARA METANÁLISIS).
La heterogeneidad en los metanálisis se refiere a la variación en los resultados del estudio entre los estudios. Por ejemplo, las medidas clásicas de heterogeneidad son:

- La Q de Cochran: se utiliza para determinar si existen diferencias entre los estudios primarios o si la variación observada se debe al azar. Una Q de p <0,10 (no 0,05) indica la presencia de heterogeneidad.
- El valor I2: cuantifica el efecto de la heterogeneidad y no depende del número de estudios o del tipo de datos de resultado. Los valores oscilan entre 0% y 100%, y representan la proporción de variabilidad entre estudios que se puede atribuir a la heterogeneidad más que al azar.
- T2 y *Tau*: son medidas de la dispersión de los tamaños del efecto verdadero entre los estudios en términos de la escala del tamaño del efecto.
- L'Abbé plot: útil para identificar no sólo los estudios que tienen resultados diferentes de otros estudios, sino también los brazos del estudio que son responsables de tales diferencias.

27. Calidad de los artículos revisados. *

28. Limitaciones generales *

29. Recomendaciones generales *

30. **Comentarios adicionales (Optativo).**
Importante: Cuando se trate de una revisión que considera a la lactancia como un outcome adicional, colocar acá el n de artículos primarios que estudiaron la lactancia, sus diseños metodológicos, y el n total de mujeres, y la calidad.

Por ejemplo:
Del total de artículos revisados, 6 ensayos clínicos aleatorizados (n = 420) y 2 estudios de cohorte (n = 120) evaluaron el abandono de la lactancia. De estos, 7 son de calidad baja y 1 de calidad alta.

Valoración de la calidad metodológica

31. ¿La pregunta de revisión está clara y explícita? *

Marca solo un óvalo.

- Si
- No
- Poco claro
- No aplica

32. ¿Fueron los criterios de inclusión apropiados para la pregunta de la revisión? *

Marca solo un óvalo.

- Si
- No
- Poco claro
- No aplica

33. ¿Fue adecuada la estrategia de búsqueda? *

Marca solo un óvalo.

- Si
- No
- Poco claro
- No aplica

34. ¿Fueron adecuadas las fuentes y los recursos utilizados para la búsqueda de estudios? *

Marca solo un óvalo.

- Si
- No
- Poco claro
- No aplica

35. ¿Son adecuados los criterios de valoración de los estudios? *

Marca solo un óvalo.

- Si
- No
- Poco claro
- No aplica

36. ¿La evaluación crítica fue realizada por dos o más revisores de forma independiente? *

Marca solo un óvalo.

- Si
- No
- Poco claro
- No aplica

37. ¿Existen métodos para minimizar los errores en la extracción de datos? *

Marca solo un óvalo.

- Si
- No
- Poco claro
- No aplica

38. ¿Fueron adecuados los métodos utilizados para combinar los estudios? *

Marca solo un óvalo.

- Si
- No
- Poco claro
- No aplica

39. ¿Se evaluó la probabilidad de sesgo de publicación? *

Marca solo un óvalo.

- Si
- No
- Poco claro
- No aplica

40. ¿Las recomendaciones para políticas y/o prácticas están respaldadas por los datos reportados? *

Marca solo un óvalo.

- Si
- No
- Poco claro
- No aplica

41. ¿Fueron adecuadas las direcciones específicas para nuevas investigaciones? *

Marca solo un óvalo.

- Si
- No
- Poco claro
- No aplica

42. Comentarios adicionales (opcional)

Herramienta de evaluación de la calidad: HEALTH EVIDENCE

43. ¿Tienen los autores una pregunta claramente enfocada [población, intervención (estrategia) y resultado(s)]? *

Marca solo un óvalo.

- Si
- No

44. ¿Se utilizaron criterios de inclusión adecuados para seleccionar los estudios primarios? *

Marca solo un óvalo.

- Si
- No

45. ¿Describieron los autores una estrategia de búsqueda exhaustiva? *

Marca solo un óvalo.

- Si
- No

46. ¿La estrategia de búsqueda ha cubierto un número adecuado de años? *

Marca solo un óvalo.

- Si
- No

47. ¿Describieron los autores el nivel de evidencia de los estudios primarios incluidos en la revisión? *

- Nivel I = ECAs solamente
- Nivel II = ECNAs, Cohortes, Casos y controles
- Nivel III = Estudios no controlados

Marca solo un óvalo.

- Si
- No

48. ¿La revisión evaluó la calidad metodológica de los estudios primarios?. Requisito mínimo: 4/7 de los siguientes: *
- Diseño de la investigación
- Muestra del estudio
- Tasas de participación
- Fuentes de sesgo (factores de confusión, sesgo del encuestado)
- Recogida de datos (medición de variables independientes/dependientes)
- Tasas de seguimiento/abandono
- Análisis de los datos

Marca solo un óvalo.

- Si
- No

49. ¿Son transparentes los resultados de la revisión? *

Marca solo un óvalo.

- Si
- No

50. ¿Fue apropiado combinar los resultados de los distintos estudios? *

Marca solo un óvalo.

- Si
- No

51. ¿Se utilizaron métodos adecuados para combinar o comparar los resultados entre los estudios? *

Marca solo un óvalo.

- Si
- No

52. ¿Apoyan los datos la interpretación del autor? *

Marca solo un óvalo.

- Si
- No

Riesgo de sesgo

53. DOMINIO 1: CRITERIOS DE ELEGIBILIDAD DEL ESTUDIO. *
Describir los criterios de elegibilidad del estudio, las restricciones de elegibilidad y si hay evidencia de que los objetivos y los criterios de elegibilidad estaban preespecificados:

Marca solo un óvalo por fila.

	Si	Probablemente si	Probablemente no	No	Sin información
**¿Se ajustó la revisión a los objetivos y criterios de elegibilidad predefinidos?**					
**¿Fueron los criterios de elegibilidad apropiados para la pregunta de revisión?**					
**¿Eran inequívocos (no ambiguos) los criterios de elegibilidad?**					
**¿Fueron apropiadas todas las restricciones en los criterios de elegibilidad basados en las características del estudio (por ejemplo, fecha, tamaño de la muestra, calidad del estudio, resultados medidos)?**					
**¿Fueron apropiadas las restricciones en los criterios de elegibilidad basados en las fuentes de información (por ejemplo, estado o formato de publicación, idioma, disponibilidad de datos)?**					

54. DOMINIO 2: IDENTIFICACIÓN Y SELECCIÓN DE ESTUDIOS. *
Describir los métodos de identificación y selección de estudios (por ejemplo, el número de revisores implicados):

Marca solo un óvalo por fila.

	Si	Probablemente si	Probablemente no	No	Sin información
**¿Incluyó la búsqueda una gama adecuada de bases de datos/fuentes electrónicas para estudios publicados y no publicados?**	⬭	⬭	⬭	⬭	⬭
**¿Se utilizaron métodos adicionales a la búsqueda en bases de datos para identificar informes relevantes?**	⬭	⬭	⬭	⬭	⬭
**¿Los términos y la estructura de la estrategia de búsqueda permitieron recuperar el mayor número posible de estudios elegibles?**	⬭	⬭	⬭	⬭	⬭
**¿Fueron apropiadas las restricciones basadas en la fecha, el formato de publicación o el idioma?**	⬭	⬭	⬭	⬭	⬭
**¿Se hizo un esfuerzo por minimizar los errores en la selección de los estudios? (p. ej., selección entre dos o más autores, resolución por consenso de discrepancias, etc.)**	⬭	⬭	⬭	⬭	⬭

55. DOMINIO 3: RECOGIDA DE DATOS Y EVALUACIÓN DE ESTUDIOS. *
Describir los métodos de recogida de datos, qué datos se extrajeron de los estudios o se recogieron por otros medios, cómo se evaluó el riesgo de sesgo (por ejemplo, el número de revisores implicados) y la herramienta utilizada para evaluar el riesgo de sesgo:

Marca solo un óvalo por fila.

	Si	Probablemente si	Probablemente no	No	Sin información
**¿Se ha procurado minimizar los errores en la recogida de datos?**					
**¿Había suficientes características de los estudios para que tanto los autores de la revisión como los lectores pudieran interpretar los resultados?**					
**¿Se recogieron todos los resultados de los estudios pertinentes para utilizarlos en la síntesis?**					
**¿Se evaluó formalmente el riesgo de sesgo (o la calidad metodológica) con criterios adecuados?**					
**¿Se hicieron esfuerzos para minimizar el error en la evaluación del riesgo de sesgo? (p. ej., evaluación por dos o más autores, resolución por consenso de discrepancias, etc.)**					

56. DOMINIO 4: SÍNTESIS Y RESULTADOS. *
Describir los métodos de síntesis:

Marca solo un óvalo por fila.

	Si	Probablemente si	Probablemente no	No	Sin información
**¿Incluyó la síntesis todos los estudios que debía?**					
**¿Se informó de todos los análisis predefinidos o se explicaron las excepciones?**					
**¿Fue adecuada la síntesis dada la naturaleza y la similitud de las preguntas de investigación, los diseños de los estudios y los resultados de los estudios incluidos?**					
**¿La variación entre estudios (heterogeneidad) fue mínima o abordado en la síntesis?**					
**¿Fueron los hallazgos robustos, por ejemplo, se demostró a través del gráfico de embudo o los análisis de sensibilidad en el caso de meta-análisis?**					
**¿Los sesgos en los estudios primarios fueron mínimos o se abordaron en la síntesis?**					

57. Valorar el riesgo de sesgo respecto a los 4 dominios anteriores. *

Si las respuestas a todas las preguntas para un dominio son "sí" o "probablemente sí", entonces el nivel de preocupación por sesgo se considera bajo. Si se responde “no” o “probablemente no” a alguna pregunta, existe la posibilidad de que exista un sesgo. La categoría “sin información” debe usarse solo cuando se informan datos insuficientes para permitir un juicio.

Marca solo un óvalo por fila.

	Bajo	Alto	No claro
**Dominio 1**			
**Dominio 2**			
**Dominio 3**			
**Dominio 4**			

58. RIESGO DE SESGO EN LA REVISIÓN *
Describir si las conclusiones están respaldadas por las evidencias:

Marca solo un óvalo por fila.

	Si	Probablemente si	Probablemente no	No	Sin información
**¿La interpretación de los resultados aborda todos los problemas identificados en los dominios 1 a 4?**	⬭	⬭	⬭	⬭	⬭
**¿Se consideró adecuadamente la relevancia de los estudios identificados para la pregunta de investigación de la revisión?**	⬭	⬭	⬭	⬭	⬭
**¿Evitaron los revisores destacar los resultados en función de su significación estadística?**	⬭	⬭	⬭	⬭	⬭

Este contenido no ha sido creado ni aprobado por Google.

Google Formularios
